# Statistical Divergences between Densities of Truncated Exponential Families with Nested Supports: Duo Bregman and Duo Jensen Divergences

**DOI:** 10.3390/e24030421

**Published:** 2022-03-17

**Authors:** Frank Nielsen

**Affiliations:** Sony Computer Science Laboratories, Tokyo 141-0022, Japan; frank.nielsen.x@gmail.com

**Keywords:** exponential family, statistical divergence, truncated exponential family, truncated normal distributions

## Abstract

By calculating the Kullback–Leibler divergence between two probability measures belonging to different exponential families dominated by the same measure, we obtain a formula that generalizes the ordinary Fenchel–Young divergence. Inspired by this formula, we define the duo Fenchel–Young divergence and report a majorization condition on its pair of strictly convex generators, which guarantees that this divergence is always non-negative. The duo Fenchel–Young divergence is also equivalent to a duo Bregman divergence. We show how to use these duo divergences by calculating the Kullback–Leibler divergence between densities of truncated exponential families with nested supports, and report a formula for the Kullback–Leibler divergence between truncated normal distributions. Finally, we prove that the skewed Bhattacharyya distances between truncated exponential families amount to equivalent skewed duo Jensen divergences.

## 1. Introduction

### 1.1. Exponential Families

Let (X,Σ) be a measurable space, and consider a regular minimal exponential family [1] E of probability measures $P_{\theta }$ all dominated by a base measure μ ($P_{\theta }\ll \mu$):

(1) $$E=\{P_{\theta }\;:\;\theta \in \Theta \}.$$

The Radon–Nikodym derivatives or densities of the probability measures $P_{\theta }$ with respect to μ can be written canonically as

(2) $$p_{\theta }\left(x\right)=\frac{dP_{\theta }}{d\mu }\left(x\right)=\exp\left(\theta^{⊤}t\left(x\right)-F\left(\theta \right)+k\left(x\right)\right),$$

where θ denotes the natural parameter, t(x) the sufficient statistic [1,2,3,4], and F(θ) the log-normalizer [1] (or cumulant function). The optional auxiliary term k(x) allows us to change the base measure μ into the measure ν such that $\frac{d\nu }{d\mu }\left(x\right)=e^{k(x)}$. The order *D* of the family is the dimension of the natural parameter space Θ:

(3) $$\Theta =\left\{\theta \in R^{D}\;:\;\int_{X}\exp\left(\theta^{⊤}t\left(x\right)+k\left(x\right)\right)\;d\mu \left(x\right)<\infty \right\},$$

where R denotes the set of reals. The sufficient statistic $t\left(x\right)=(t_{1}\left(x\right),\ldots ,t_{D}\left(x\right))$ is a vector of *D* functions. The sufficient statistic t(x) is said to be minimal when the D+1 functions 1, $t_{1}\left(x\right)$, *…*, $t_{D}\left(x\right)$ are linearly independent [1]. The sufficient statistics t(x) are such that the probability Pr[X|θ]=Pr[X|t(X)]. That is, all information necessary for the statistical inference of parameter θ is contained in t(X). Exponential families are characterized as families of parametric distributions with finite-dimensional sufficient statistics [1]. Exponential families $\left\{p_{\lambda }\right\}$ include among others the exponential, normal, gamma/beta, inverse gamma, inverse Gaussian, and Wishart distributions once a reparameterization θ=θ(λ) of the parametric distributions $\left\{p_{\lambda }\right\}$ is performed to reveal their natural parameters [1].

When the sufficient statistic t(x) is *x*, these exponential families [1] are called natural exponential families or tilted exponential families [5] in the literature. Indeed, the distributions $P_{\theta }$ of the exponential family E can be interpreted as distributions obtained by tilting the base measure μ [6]. In this paper, we consider either discrete exponential families like the family of Poisson distributions (univariate distributions of order D=1 with respect to the counting measure) or continuous exponential families like the family of normal distributions (univariate distributions of order D=2 with respect to the Lebesgue measure). The Radon–Nikodym derivative of a discrete exponential family is a probability mass function (pmf), and the Radon–Nikodym derivative of a continuous exponential family is a probability density function (pdf). The support of a pmf p(x) is supp(p)={x∈Z:p(x)>0} (where Z denotes the set of integers) and the support of a *d*-variate pdf p(x) is $\mathrm{supp}\left(p\right)=\{x\in R^{d}\;:\;p\left(x\right)>0\}$. The Poisson distributions have support N∪{0} where N denotes the set of natural numbers {1,2,…,}. Densities of an exponential family all have coinciding support [1].

### 1.2. Truncated Exponential Families with Nested Supports

In this paper, we shall consider truncated exponential families [7] with nested supports. A truncated exponential family is a set of parametric probability distributions obtained by truncation of the support of an exponential family. Truncated exponential families are exponential families but their statistical inference is more subtle [8,9]. Let $E_{\mathrm{Trunc}}=\left\{q_{\theta }\right\}$ be a truncated exponential family of $E=\{p_{\theta }\}$ with nested supports $\mathrm{supp}\left(q_{\theta }\right)\subset \mathrm{supp}\left(p_{\theta }\right)$. The canonical decompositions of densities $p_{\theta }$ and $q_{\theta }$ have the following expressions:

(4) $$\begin{array}{ccc} p_{\theta }\left(x\right) & = & \exp\left(\theta^{⊤}t\left(x\right)+k\left(x\right)-F\left(\theta \right)\right), \end{array}$$

(5) $$\begin{array}{ccc} q_{\theta }\left(x\right) & = & \frac{p_{\theta }\left(x\right)}{Z^{X_{\mathrm{Trunc}}}\left(\theta \right)}=\exp\left(\theta^{⊤}t\left(x\right)+k\left(x\right)-F_{\mathrm{Trunc}}\left(\theta \right)\right), \end{array}$$

where the log-normalizer of the truncated exponential family is:

(6) $$F_{\mathrm{Trunc}}\left(\theta \right)=F\left(\theta \right)+\log Z^{X_{\mathrm{Trunc}}}\left(\theta \right),$$

where $Z^{X_{\mathrm{Trunc}}}\left(\theta \right)$ is a normalizing term that takes into account the truncated support $X_{\mathrm{Trunc}}$. These equations show that densities of truncated exponential families only differ by their log-normalizer functions. Let $X_{\mathrm{Trunc}}$ denote the support of the distributions of $E_{\mathrm{Trunc}}=\mathrm{supp}\left(q_{\theta }\right)$ and $X=\mathrm{supp}(p_{\theta })$ the support of E. Family $E_{\mathrm{Trunc}}$ is a truncated exponential family of E that can be notationally written as $E_{X_{\mathrm{Trunc}}}$. Family E can also be interpreted as the (un)truncated exponential family $E_{X}$ with densities $p_{\theta }^{X}=p_{\theta }$. A truncated exponential family $E_{X_{\mathrm{Trunc}}}$ of E is said to have nested support when $X_{\mathrm{Trunc}}\subset X$. For example, the family of half-normal distributions defined on the support $X_{\mathrm{Trunc}}=\left[0,\infty \right)$ is a nested truncated exponential family of the family of normal distributions defined on the support X=(−∞,∞).

### 1.3. Kullback–Leibler Divergence between Exponential Family Distributions

For two σ-finite probability measures *P* and *Q* on (X,Σ) such that *P* is dominated by *Q* (P≪Q), the Kullback–Leibler divergence between *P* and *Q* is defined by

(7) $$D_{\mathrm{KL}}\left[P:Q\right]=\int_{X}\log\frac{dP}{dQ}\;dP=E_{P}\left[\log\frac{dP}{dQ}\right],$$

where $E_{P}\left[X\right]$ denotes the expectation of a random variable X∼P [10]. When P≪Q, we set $D_{\mathrm{KL}}\left[P:Q\right]=+\infty$. Gibbs’ inequality [11] $D_{\mathrm{KL}}\left[P:Q\right]\geq 0$ shows that the Kullback–Leibler divergence (KLD for short) is always non-negative. The proof of Gibbs’ inequality relies on Jensen’s inequality and holds for the wide class of *f*-divergences [12] induced by convex generators f(u):

(8) $$I_{f}\left[P:Q\right]=\int_{X}f\left(\frac{dQ}{dP}\right)\;dP\geq f\left(\int_{X}\frac{dQ}{dP}\;dP\right)\geq f\left(1\right).$$

The KLD is an *f*-divergence obtained for the convex generator f(u)=−logu.

### 1.4. Kullback–Leibler Divergence between Exponential Family Densities

It is well-known that the KLD between two distributions $P_{\theta_{1}}$ and $P_{\theta_{2}}$ of E amounts to computing an equivalent Fenchel–Young divergence [13]:

(9) $$D_{\mathrm{KL}}\left[P_{\theta_{1}}:P_{\theta_{2}}\right]=\int_{X}p_{\theta_{1}}\left(x\right)\log\frac{p_{\theta_{1}}\left(x\right)}{p_{\theta_{2}}\left(x\right)}\;d\mu \left(x\right)=Y_{F,F^{*}}\left(\theta_{2},\eta_{1}\right),$$

where $\eta =\nabla F\left(\theta \right)=E_{P_{\theta }}\left[t\left(x\right)\right]$ is the moment parameter [1] and

(10) $$\nabla F\left(\theta \right)={\left[\frac{\partial }{\partial \theta_{1}}F\left(\theta \right),\ldots ,\frac{\partial }{\partial \theta_{D}}F\left(\theta \right)\right]}^{⊤},$$

is the gradient of *F* with respect to $\theta ={\left[\theta_{1},\ldots ,\theta_{D}\right]}^{⊤}$. The Fenchel–Young divergence is defined for a pair of strictly convex conjugate functions [14] F(θ) and $F^{*}\left(\eta \right)$ related by the Legendre–Fenchel transform by

(11) $$Y_{F,F^{*}}\left(\theta_{1},\eta_{2}\right):=F\left(\theta_{1}\right)+F^{*}\left(\eta_{2}\right)-\theta_{1}^{⊤}\eta_{2}.$$

Amari (1985) first introduced this formula as the canonical divergence of dually flat spaces in information geometry [15] (Equation 3.21), and proved that the Fenchel–Young divergence is obtained as the KLD between densities belonging to the same exponential family [15] (Theorem 3.7). Azoury and Warmuth expressed the KLD $D_{\mathrm{KL}}\left[P_{\theta_{1}}:P_{\theta_{2}}\right]$ using dual Bregman divergences in [13] (2001):

(12) $$D_{\mathrm{KL}}\left[P_{\theta_{1}}:P_{\theta_{2}}\right]=B_{F}\left(\theta_{2}:\theta_{1}\right)=B_{F^{*}}\left(\eta_{1}:\eta_{2}\right),$$

where a Bregman divergence [16] $B_{F}\left(\theta_{1}:\theta_{2}\right)$ is defined for a strictly convex and differentiable generator F(θ) by:

(13) $$B_{F}\left(\theta_{1}:\theta_{2}\right):=F\left(\theta_{1}\right)-F\left(\theta_{2}\right)-{\left(\theta_{1}-\theta_{2}\right)}^{⊤}\nabla F\left(\theta_{2}\right).$$

Acharyya termed the divergence $Y_{F,F^{*}}$ the Fenchel–Young divergence in his PhD thesis [17] (2013), and Blondel et al. called such divergences Fenchel–Young losses (2020) in the context of machine learning [18] (Equation (9) in Definition 2). This term was also used by the author the Legendre–Fenchel divergence in [19]. The Fenchel–Young divergence stems from the Fenchel–Young inequality [14,20]:

(14) $$F\left(\theta_{1}\right)+F^{*}\left(\eta_{2}\right)\geq \theta_{1}^{⊤}\eta_{2},$$

with equality if and only if $\eta_{2}=\nabla F\left(\theta_{1}\right)$.

**Figure 1:**
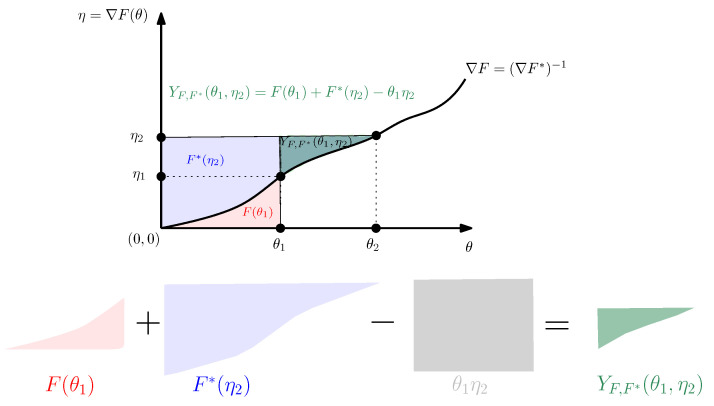
Visualizing the Fenchel–Young divergence.

Figure 1 visualizes the 1D Fenchel–Young divergence and gives a geometric proof that $Y_{F,F^{*}}\left(\theta_{1},\eta_{2}\right)\geq 0$ with equality if and only if $\eta_{2}=F^{\prime }\left(\theta_{1}\right)$. Indeed, by considering the behavior of the Legendre–Fenchel transformation under translations:

- if $F_{t}\left(\theta \right)=F\left(\theta +t\right)$ then $F_{t}^{*}\left(\eta \right)=F^{*}\left(\eta \right)-\eta^{⊤}t$ for all t∈R, and
- if $F_{\lambda }\left(\theta \right)=F\left(\theta \right)+\lambda$ then $F_{\lambda }^{*}\left(\eta \right)=F^{*}\left(\eta \right)-\lambda$ for all λ∈R,

we may assume without loss of generality that F(0)=0. The function $F^{\prime }\left(\theta \right)$ is strictly increasing and continuous since F(θ) is a strictly convex and differentiable convex function. Thus we have $F\left(\theta \right)=\int_{0}^{\theta }F^{\prime }\left(\theta \right)\;d\theta$ and $F^{*}\left(\eta \right)=\int_{0}^{\eta }{F^{*}}^{\prime }\left(\eta \right)\;d\eta =\int_{0}^{\eta }{F^{\prime }}^{-1}\left(\eta \right)\;d\eta$.

The Bregman divergence $B_{F}\left(\theta_{1}:\theta_{2}\right)$ amounts to a dual Bregman divergence [13] between the dual parameters with swapped order: $B_{F}\left(\theta_{1}:\theta_{2}\right)=B_{F^{*}}\left(\eta_{2}:\eta_{1}\right)$ where $\eta_{i}=\nabla F\left(\theta_{i}\right)$ for i∈{1,2}. Thus the KLD between two distributions $P_{\theta_{1}}$ and $P_{\theta_{2}}$ of E can be expressed equivalently as follows:

(15) $$D_{\mathrm{KL}}\left[P_{\theta_{1}}:P_{\theta_{2}}\right]=Y_{F,F^{*}}\left(\theta_{2}:\eta_{1}\right)=B_{F}\left(\theta_{2}:\theta_{1}\right)=B_{F^{*}}\left(\eta_{1}:\eta_{2}\right)=Y_{F^{*},F}\left(\eta_{1}:\eta_{2}\right).$$

The symmetrized Kullback–Leibler divergence $D_{J}\left[P_{\theta_{1}}:P_{\theta_{2}}\right]$ between two distributions $P_{\theta_{1}}$ and $P_{\theta_{2}}$ of E is called Jeffreys’ divergence [21] and amounts to a symmetrized Bregman divergence [22]:

(16) $$\begin{array}{ccc} D_{J}\left[P_{\theta_{1}}:P_{\theta_{2}}\right] & = & D_{\mathrm{KL}}\left[P_{\theta_{1}}:P_{\theta_{2}}\right]+D_{\mathrm{KL}}\left[P_{\theta_{2}}:P_{\theta_{1}}\right], \end{array}$$

(17) $$\begin{array}{ccc} & = & B_{F}\left(\theta_{2}:\theta_{1}\right)+B_{F}\left(\theta_{1}:\theta_{2}\right), \end{array}$$

(18) $$\begin{array}{ccc} & = & {\left(\theta_{2}-\theta_{1}\right)}^{⊤}\left(\eta_{2}-\eta_{1}\right):=S_{F}\left(\theta_{1},\theta_{2}\right). \end{array}$$

Note that the Bregman divergence $B_{F}\left(\theta_{1}:\theta_{2}\right)$ can also be interpreted as a surface area:

(19) $$B_{F}\left(\theta_{1}:\theta_{2}\right)=\int_{\theta_{2}}^{\theta_{1}}\left(F^{\prime }\left(\theta \right)-F^{\prime }\left(\theta_{2}\right)\right)d\theta .$$

**Figure 2:**
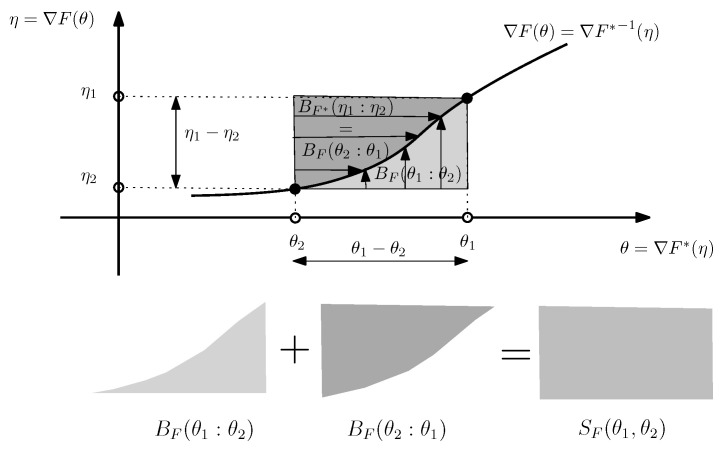
Visualizing the sided and symmetrized Bregman divergences.

Figure 2 illustrates the sided and symmetrized Bregman divergences.

### 1.5. Contributions and Paper Outline

We recall in Section 2 the formula obtained for the Kullback–Leibler divergence between two exponential family densities equivalent to each other [23] (Equation (29)). Inspired by this formula, we give a definition of the duo Fenchel–Young divergence induced by a pair of strictly convex functions $F_{1}$ and $F_{2}$ (Definition 1) in Section 3, and prove that the divergence is always non-negative provided that $F_{1}$ upper bounds $F_{2}$. We then define the duo Bregman divergence (Definition 2) corresponding to the duo Fenchel–Young divergence. In Section 4, we show that the Kullback–Leibler divergence between a truncated density and a density of a same parametric exponential family amounts to a duo Fenchel–Young divergence or equivalently to a duo Bregman divergence on swapped parameters (Theorem 1). That is, we consider a truncated exponential family [7] $E_{1}$ of an exponential family $E_{1}$ such that the common support of the distributions of $E_{1}$ is contained in the common support of the distributions of $E_{2}$ and both canonical decompositions of the families coincide (see Equation (2)). In particular, when $E_{2}$ is also a truncated exponential family of E, then we express the KLD between two truncated distributions as a duo Bregman divergence. As examples, we report the formula for the Kullback–Leibler divergence between two densities of truncated exponential families (Corollary 1), and illustrate the formula for the Kullback–Leibler divergence between truncated exponential distributions (Example 6) and for the Kullback–Leibler divergence between truncated normal distributions (Example 7).

In Section 5, we further consider the skewed Bhattacharyya distance between densities of truncated exponential families and prove that it amounts to a duo Jensen divergence (Theorem 2). Finally, we conclude in Section 6.

## 2. Kullback–Leibler Divergence between Different Exponential Families

Consider now two exponential families [1] P and Q defined by their Radon–Nikodym derivatives with respect to two positive measures $\mu_{P}$ and $\mu_{Q}$ on (X,Σ):

(20) $$\begin{array}{ccc} P & = & \left\{P_{\theta }\;:\;\theta \in \Theta \right\}, \end{array}$$

(21) $$\begin{array}{ccc} Q & = & \left\{Q_{\theta^{\prime }}\;:\;\theta^{\prime }\in \Theta^{\prime }\right\}. \end{array}$$

The corresponding natural parameter spaces are

(22) $$\begin{array}{ccc} \Theta & = & \left\{\theta \in R^{D}\;:\;\int_{X}\exp\left(\theta^{⊤}t_{P}\left(x\right)+k_{P}\left(x\right)\right)\;d\mu_{P}\left(x\right)<\infty \right\}, \end{array}$$

(23) $$\begin{array}{ccc} \Theta^{\prime } & = & \left\{\theta^{\prime }\in R^{D^{\prime }}\;:\;\int_{X}\exp\left({\theta^{\prime }}^{⊤}t_{Q}\left(x\right)+k_{Q}\left(x\right)\right)\;d\mu_{Q}\left(x\right)<\infty \right\}, \end{array}$$

The order of P is *D*, $t_{P}\left(x\right)$ denotes the sufficient statistics of $P_{\theta }$, and $k_{P}\left(x\right)$ is a term to adjust/tilt the base measure $\mu_{P}$. Similarly, the order of Q is $D^{\prime }$, $t_{Q}\left(x\right)$ denotes the sufficient statistics of $Q_{\theta^{\prime }}$, and $k_{Q}\left(x\right)$ is an optional term to adjust the base measure $\mu_{Q}$. Let $p_{\theta }$ and $q_{\theta^{\prime }}$ denote the Radon–Nikodym derivatives with respect to the measures $\mu_{P}$ and $\mu_{Q}$, respectively:

(24) $$\begin{array}{ccc} p_{\theta } & = & \frac{dP_{\theta }}{d\mu_{P}}=\exp\left(\theta^{⊤}t_{P}\left(x\right)-F_{P}\left(\theta \right)+k_{P}\left(x\right)\right), \end{array}$$

(25) $$\begin{array}{ccc} q_{\theta^{\prime }} & = & \frac{dQ_{\theta^{\prime }}}{d\mu_{Q}}=\exp\left({\theta^{\prime }}^{⊤}t_{Q}\left(x\right)-F_{Q}\left(\theta^{\prime }\right)+k_{Q}\left(x\right)\right), \end{array}$$

where $F_{P}\left(\theta \right)$ and $F_{Q}\left(\theta^{\prime }\right)$ denote the corresponding log-normalizers of P and Q, respectively.

(26) $$\begin{array}{ccc} F_{P}\left(\theta \right) & = & \log\left(\int \exp\left(\theta^{⊤}t_{P}\left(x\right)+k_{P}\left(x\right)\right)\;d\mu_{P}\left(x\right)\right), \end{array}$$

(27) $$\begin{array}{ccc} F_{Q}\left(\theta \right) & = & \log\left(\int \exp\left(\theta^{⊤}t_{Q}\left(x\right)+k_{Q}\left(x\right)\right)\;d\mu_{Q}\left(x\right)\right). \end{array}$$

The functions $F_{P}$ and $F_{Q}$ are strictly convex and real analytic [1]. Hence, those functions are infinitely many times differentiable on their open natural parameter spaces.

Consider the KLD between $P_{\theta }\in P$ and $Q_{\theta^{\prime }}\in Q$ such that $\mu_{P}=\mu_{Q}$ (and hence $P_{\theta }\ll Q_{\theta^{\prime }}$). Then the KLD between $P_{\theta }$ and $Q_{\theta^{\prime }}$ was first considered in [23]:

(28) $$\begin{array}{ccc} D_{\mathrm{KL}}\left[P_{\theta }:Q_{\theta^{\prime }}\right] & = & E_{P}\left[\log\left(\frac{dP_{\theta }}{dQ_{\theta^{\prime }}}\right)\right],\\ & = & E_{P_{\theta }}\left[\left(\theta^{⊤}t_{P}\left(x\right)-{\theta^{\prime }}^{⊤}t_{Q}\left(x\right)-F_{P}\left(\theta \right)+F_{Q}\left(\theta^{\prime }\right)+k_{P}\left(x\right)-k_{Q}\left(x\right)\right)\underset{=1}{\underset{︸}{\frac{d\mu_{P}}{d\mu_{Q}}}}\right],\\ & = & F_{Q}\left(\theta^{\prime }\right)-F_{P}\left(\theta \right)+\theta^{⊤}E_{P_{\theta }}\left[t_{P}\left(x\right)\right]-{\theta^{\prime }}^{⊤}E_{P_{\theta }}\left[t_{Q}\left(x\right)\right]+E_{P_{\theta }}\left[k_{P}\left(x\right)-k_{Q}\left(x\right)\right]. \end{array}$$

Recall that the dual parameterization of an exponential family density $P_{\theta }$ is $P^{\eta }$ with $\eta =E_{P_{\theta }}\left[t_{P}\left(x\right)\right]=\nabla F_{P}\left(\theta \right)$ [1], and that the Fenchel–Young equality is $F\left(\theta \right)+F^{*}\left(\eta \right)=\theta^{⊤}\eta$ for η=∇F(θ). Thus the KLD between $P_{\theta }$ and $Q_{\theta^{\prime }}$ can be rewritten as

(29) $$D_{\mathrm{KL}}\left[P_{\theta }:Q_{\theta^{\prime }}\right]=F_{Q}\left(\theta^{\prime }\right)+F_{P}^{*}\left(\eta \right)-{\theta^{\prime }}^{⊤}E_{P_{\theta }}\left[t_{Q}\left(x\right)\right]+E_{P_{\theta }}\left[k_{P}\left(x\right)-k_{Q}\left(x\right)\right].$$

This formula was reported in [23] and generalizes the Fenchel–Young divergence [17] obtained when P=Q (with $t_{P}\left(x\right)=t_{Q}\left(x\right)$, $k_{P}\left(x\right)=k_{Q}\left(x\right)$, and $F\left(\theta \right)=F_{P}\left(\theta \right)=F_{Q}\left(\theta \right)$ and $F^{*}\left(\eta \right)=F_{P}^{*}\left(\eta \right)=F_{Q}^{*}\left(\eta \right)$).

The formula of Equation (29) was illustrated in [23] with two examples: the KLD between Laplacian distributions and zero-centered Gaussian distributions, and the KLD between two Weibull distributions. Both these examples use the Lebesgue base measure for $\mu_{P}$ and $\mu_{Q}$.

Let us report another example that uses the counting measure as the base measure for $\mu_{P}$ and $\mu_{Q}$.

**Example** **1.** *Consider the KLD between a Poisson probability mass function (pmf) and a geometric pmf. The canonical decompositions of the Poisson and geometric pmfs are summarized in Table 1. The KLD between a Poisson pmf $p_{\lambda }$ and a geometric pmf $q_{p}$ is equal to*

(30) $$\begin{array}{ccc} D_{\mathrm{KL}}\left[P_{\lambda }:Q_{p}\right] & = & F_{Q}\left(\theta^{\prime }\right)+F_{P}^{*}\left(\eta \right)-E_{P_{\theta }}\left[t_{Q}\left(x\right)\right]\cdot \theta^{\prime }+E_{P_{\theta }}\left[k_{P}\left(x\right)-k_{Q}\left(x\right)\right], \end{array}$$

(31) $$\begin{array}{ccc} & = & -\log p+\lambda \log\lambda -\lambda -\lambda \log\left(1-p\right)-E_{P_{\lambda }}\left[\log x!\right] \end{array}$$

*Since $E_{p_{\lambda }}\left[-\log x!\right]=-\sum_{k=0}^{\infty }e^{-\lambda }\frac{\lambda^{k}\log\left(k!\right)}{k!}$, we have*

(32) $$D_{\mathrm{KL}}\left[P_{\lambda }:Q_{p}\right]=-\log p+\lambda \log\frac{\lambda }{1-p}-\lambda -\sum_{k=0}^{\infty }e^{-\lambda }\frac{\lambda^{k}\log\left(k!\right)}{k!}.$$

*Note that we can calculate the KLD between two geometric distributions $Q_{p_{1}}$ and $Q_{p_{2}}$ as*

(33) $$\begin{array}{ccc} D_{\mathrm{KL}}\left[Q_{p_{1}}:Q_{p_{2}}\right] & = & B_{F_{Q}}\left(\theta \left(p_{2}\right):\theta \left(p_{1}\right)\right), \end{array}$$

(34) $$\begin{array}{ccc} & = & F_{Q}\left(\theta \left(p_{2}\right)\right)-F_{Q}\left(\theta \left(p_{1}\right)\right)-\left(\theta \left(p_{2}\right)-\theta \left(p_{1}\right)\right)\eta \left(p_{1}\right), \end{array}$$

*We obtain:*

$$D_{\mathrm{KL}}\left[Q_{p_{1}}:Q_{p_{2}}\right]=\log\left(\frac{p_{1}}{p_{2}}\right)-\left(1-\frac{1}{p_{1}}\right)\log\frac{1-p_{1}}{1-p_{2}}.$$

## 3. The Duo Fenchel–Young Divergence and Its Corresponding Duo Bregman Divergence

Inspired by formula of Equation (29), we shall define the *duo Fenchel–Young divergence* using a *dominance condition* on a pair $\left(F_{1}\left(\theta \right),F_{2}\left(\theta \right)\right)$ of strictly convex generators.

**Definition** **1** (duo Fenchel–Young divergence)**.** Let $F_{1}\left(\theta \right)$ and $F_{2}\left(\theta \right)$ be two strictly convex functions such that $F_{1}\left(\theta \right)\geq F_{2}\left(\theta \right)$ for any $\theta \in \Theta_{12}=\mathrm{dom}\left(F_{1}\right)\cap \mathrm{dom}\left(F_{2}\right)$. Then the duo Fenchel–Young divergence $Y_{F_{1},F_{2}^{*}}\left(\theta ,\eta^{\prime }\right)$ is defined by

(35) $$Y_{F_{1},F_{2}^{*}}\left(\theta ,\eta^{\prime }\right):=F_{1}\left(\theta \right)+F_{2}^{*}\left(\eta^{\prime }\right)-\theta^{⊤}\eta^{\prime }.$$

When $F_{1}\left(\theta \right)=F_{2}\left(\theta \right)=:F\left(\theta \right)$, we have $F_{1}^{*}\left(\eta \right)=F_{2}^{*}\left(\eta \right)=:F^{*}\left(\eta \right)$, and we retrieve the ordinary Fenchel–Young divergence [17]:

(36) $$Y_{F,F^{*}}\left(\theta ,\eta^{\prime }\right):=F\left(\theta \right)+F^{*}\left(\eta^{\prime }\right)-\theta^{⊤}\eta^{\prime }\geq 0.$$

Note that in Equation (35), we have $\eta^{\prime }=\nabla F_{2}\left(\theta^{\prime }\right)$.

**Property** **1** (Non-negative duo Fenchel–Young divergence)**.** *The duo Fenchel–Young divergence is always non-negative.*

**Proof.**  The proof relies on the reverse dominance property of strictly convex and differentiable conjugate functions:

**Lemma** **1** (Reverse majorization order of functions by the Legendre–Fenchel transform)**.** Let $F_{1}\left(\theta \right)$ and $F_{2}\left(\theta \right)$ be two Legendre-type convex functions [14]. Then if $F_{1}\left(\theta \right)\geq F_{2}\left(\theta \right)$ then we have $F_{2}^{*}\left(\eta \right)\geq F_{1}^{*}\left(\eta \right)$.

**Figure 3:**
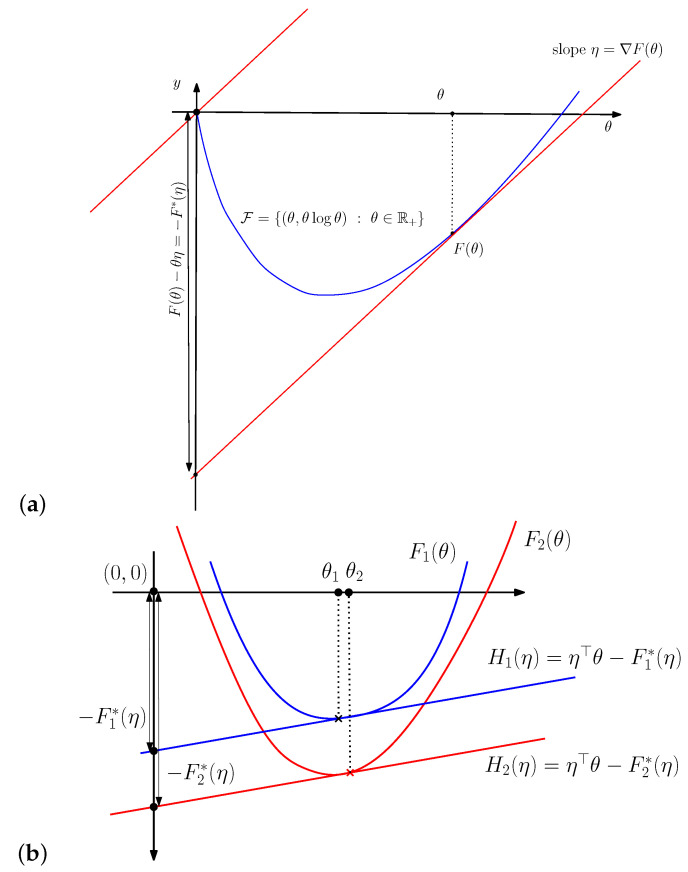
(**a**) Visual illustration of the Legendre–Fenchel transformation: $F^{*}\left(\eta \right)$ is measured as the vertical gap (left long black line with both arrows) between the origin and the hyperplane of the “slope” η tangent at F(θ) evaluated at θ=0. (**b**) The Legendre transforms $F_{1}^{*}\left(\eta \right)$ and $F_{1}^{*}\left(\eta \right)$ of two functions $F_{1}\left(\theta \right)$ and $F_{2}\left(\theta \right)$ such that $F_{1}\left(\theta \right)\geq F_{2}\left(\theta \right)$ reverse the dominance order: $F_{2}^{*}\left(\eta \right)\geq F_{1}^{*}\left(\eta \right)$.

**Proof** **.** This property is graphically illustrated in Figure 3. The reverse dominance property of the Legendre–Fenchel transformation can be checked algebraically as follows:

(37) $$\begin{array}{ccc} F_{1}^{*}\left(\eta \right) & = & \underset{\theta \in \Theta }{\sup}\left\{\eta^{⊤}\theta -F_{1}\left(\theta \right)\right\}, \end{array}$$

(38) $$\begin{array}{ccc} & = & \eta^{⊤}\theta_{1}-F_{1}\left(\theta_{1}\right)\;\;\left(\mathrm{with}\eta =\nabla F_{1}\left(\theta_{1}\right)\right), \end{array}$$

(39) $$\begin{array}{ccc} & \leq & \eta^{⊤}\theta_{1}-F_{2}\left(\theta_{1}\right), \end{array}$$

(40) $$\begin{array}{ccc} & \leq & \underset{\theta \in \Theta }{\sup}\left\{\eta^{⊤}\theta -F_{2}\left(\theta \right)\right\}=F_{2}^{*}\left(\eta \right). \end{array}$$

 □

Thus we have $F_{1}^{*}\left(\eta \right)\leq F_{2}^{*}\left(\eta \right)$ when $F_{1}\left(\theta \right)\geq F_{2}\left(\theta \right)$. Therefore it follows that $Y_{F_{1},F_{2}^{*}}\left(\theta ,\eta^{\prime }\right)$≥0 since we have

(41) $$\begin{array}{ccc} Y_{F_{1},F_{2}^{*}}\left(\theta ,\eta^{\prime }\right) & := & F_{1}\left(\theta \right)+F_{2}^{*}\left(\eta^{\prime }\right)-\theta^{⊤}\eta^{\prime }, \end{array}$$

(42) $$\begin{array}{ccc} & \geq & F_{1}\left(\theta \right)+F_{1}^{*}\left(\eta^{\prime }\right)-\theta^{⊤}\eta^{\prime }=Y_{F_{1},F_{1}^{*}}\left(\theta ,\eta^{\prime }\right)\geq 0, \end{array}$$

where $Y_{F_{1},F_{1}^{*}}$ is the ordinary Fenchel–Young divergence, which is guaranteed to be non-negative from the Fenchel–Young inequality. □

**Figure 4:**
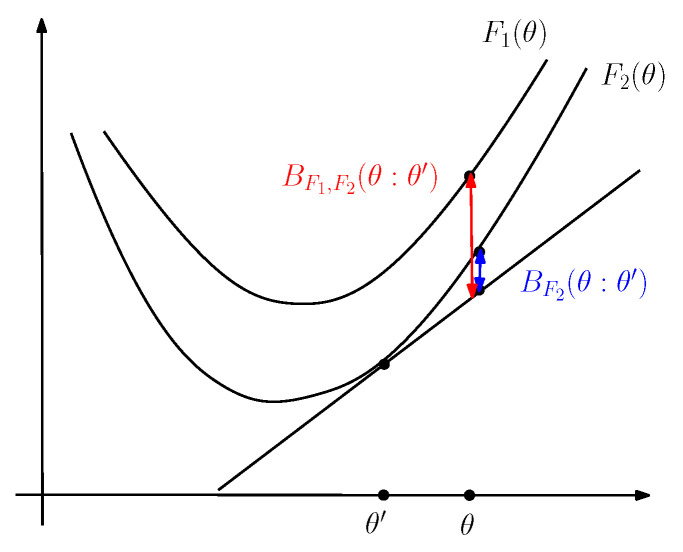
The duo Bregman divergence induced by two strictly convex and differentiable functions $F_{1}$ and $F_{2}$ such that $F_{1}\left(\theta \right)\geq F_{2}\left(\theta \right)$. We check graphically that $B_{F_{1},F_{2}}\left(\theta :\theta^{\prime }\right)\geq B_{F_{2}}\left(\theta :\theta^{\prime }\right)$ (vertical gaps).

We can express the duo Fenchel–Young divergence using the primal coordinate systems as a generalization of the Bregman divergence to two generators that we term the duo Bregman divergence (see Figure 4):

(43) $$B_{F_{1},F_{2}}\left(\theta :\theta^{\prime }\right):=Y_{F_{1},F_{2}^{*}}\left(\theta ,\eta^{\prime }\right)=F_{1}\left(\theta \right)-F_{2}\left(\theta^{\prime }\right)-{\left(\theta -\theta^{\prime }\right)}^{⊤}\nabla F_{2}\left(\theta^{\prime }\right),$$

with $\eta^{\prime }=\nabla F_{2}\left(\theta^{\prime }\right)$.

This generalized Bregman divergence is non-negative when $F_{1}\left(\theta \right)\geq F_{2}\left(\theta \right)$. Indeed, we check that

(44) $$\begin{array}{ccc} B_{F_{1},F_{2}}\left(\theta :\theta^{\prime }\right) & = & F_{1}\left(\theta \right)-F_{2}\left(\theta^{\prime }\right)-{\left(\theta -\theta^{\prime }\right)}^{⊤}\nabla F_{2}\left(\theta^{\prime }\right), \end{array}$$

(45) $$\begin{array}{ccc} & \geq & F_{2}\left(\theta \right)-F_{2}\left(\theta^{\prime }\right)-{\left(\theta -\theta^{\prime }\right)}^{⊤}\nabla F_{2}\left(\theta^{\prime }\right)=B_{F_{2}}\left(\theta :\theta^{\prime }\right)\geq 0. \end{array}$$

**Definition** **2** (duo Bregman divergence)**.** *Let $F_{1}\left(\theta \right)$ and $F_{2}\left(\theta \right)$ be two strictly convex functions such that $F_{1}\left(\theta \right)\geq F_{2}\left(\theta \right)$ for any $\theta \in \Theta_{12}=\mathrm{dom}\left(F_{1}\right)\cap \mathrm{dom}\left(F_{2}\right)$. Then the generalized Bregman divergence is defined by*

(46) $$B_{F_{1},F_{2}}\left(\theta :\theta^{\prime }\right)=F_{1}\left(\theta \right)-F_{2}\left(\theta^{\prime }\right)-{\left(\theta -\theta^{\prime }\right)}^{⊤}\nabla F_{2}\left(\theta^{\prime }\right)\geq 0.$$

**Example** **2.** *Consider $F_{1}\left(\theta \right)=\frac{a}{2}\theta^{2}$ for a>0. We have η=aθ, $\theta =\frac{\eta }{a}$, and*

(47) $$F_{1}^{*}\left(\eta \right)=\frac{\eta^{2}}{a}-\frac{a}{2}\frac{\eta^{2}}{a^{2}}=\frac{\eta^{2}}{2a}.$$

Let $F_{2}\left(\theta \right)=\frac{1}{2}\theta^{2}$ so that $F_{1}\left(\theta \right)\geq F_{2}\left(\theta \right)$ for a≥1. We check that $F_{1}^{*}\left(\eta \right)=\frac{\eta^{2}}{2a}\leq F_{2}^{*}\left(\eta \right)$ when a≥1. The duo Fenchel–Young divergence is

(48) $$Y_{F_{1},F_{2}^{*}}\left(\theta ,\eta^{\prime }\right)=\frac{a}{2}\theta^{2}+\frac{1}{2}{\eta^{\prime }}^{2}-\theta \eta^{\prime }\geq 0,$$

*when a≥1. We can express the duo Fenchel–Young divergence in the primal coordinate systems as*

(49) $$B_{F_{1},F_{2}}\left(\theta ,\theta^{\prime }\right)=\frac{a}{2}\theta^{2}+\frac{1}{2}{\theta^{\prime }}^{2}-\theta \theta^{\prime }.$$

**Figure 5:**
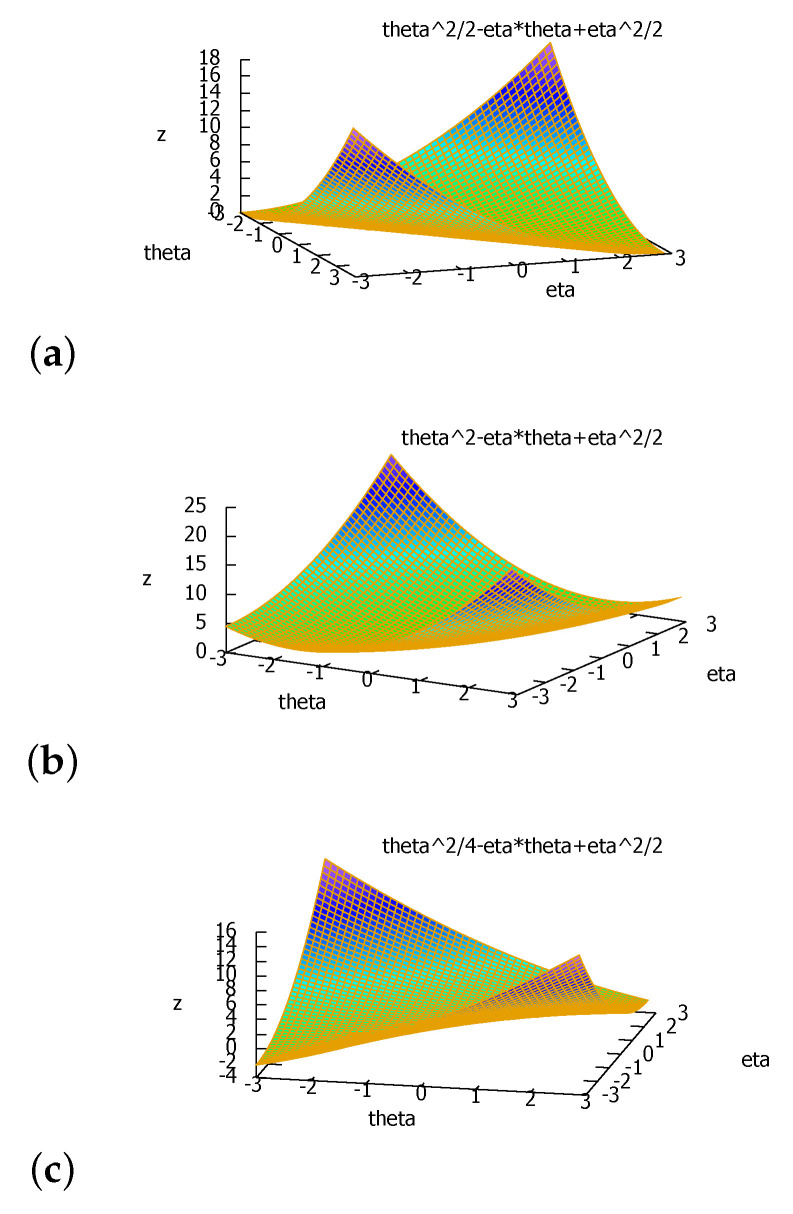
The duo half squared Euclidean distance $D_{a}^{2}\left(\theta :\theta^{\prime }\right):=\frac{a}{2}\theta^{2}+\frac{1}{2}{\theta^{\prime }}^{2}-\theta \theta^{\prime }$ is non-negative when a≥1: (**a**) half squared Euclidean distance (a=1), (**b**) a=2, (**c**) $a=\frac{1}{2}$, which shows that the divergence can be negative then since a<1.

*When a=1, $F_{1}\left(\theta \right)=F_{2}\left(\theta \right)=\frac{1}{2}\theta^{2}:=F\left(\theta \right)$, and we obtain $B_{F}\left(\theta ,\theta^{\prime }\right)=\frac{1}{2}{∥\theta -\theta^{\prime }∥}_{2}^{2}$, half the squared Euclidean distance as expected. Figure 5 displays the graph plot of the duo Bregman divergence for several values of a.*

**Figure 6:**
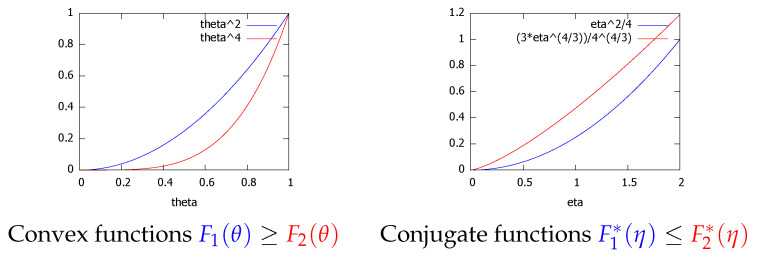
The Legendre transform reverses the dominance ordering: $F_{1}\left(\theta \right)=\theta^{2}\geq F_{2}\left(\theta \right)=\theta^{4}\Leftrightarrow F_{1}^{*}\left(\eta \right)\leq F_{2}^{*}\left(\eta \right)$ for θ∈[0,1].

**Example** **3.** Consider $F_{1}\left(\theta \right)=\theta^{2}$ and $F_{2}\left(\theta \right)=\theta^{4}$ on the domain Θ=[0,1]. We have $F_{1}\left(\theta \right)\geq F_{2}\left(\theta \right)$ for θ∈Θ. The convex conjugate of $F_{1}\left(\eta \right)$ is $F_{1}^{*}\left(\eta \right)=\frac{1}{4}\eta^{2}$. We have

(50) $$F_{2}^{*}\left(\eta \right)=\eta^{\frac{4}{3}}\left({\left(\frac{1}{4}\right)}^{\frac{1}{3}}-{\left(\frac{1}{4}\right)}^{\frac{4}{3}}\right)=\frac{3}{4^{\frac{4}{3}}}\eta^{\frac{4}{3}}$$

with $\eta_{2}\left(\theta \right)=4\theta^{3}$. Figure 6 plots the convex functions $F_{1}\left(\theta \right)$ and $F_{2}\left(\theta \right)$, and their convex conjugates $F_{1}^{*}\left(\eta \right)$ and $F_{2}^{*}\left(\eta \right)$. We observe that $F_{1}\left(\theta \right)\geq F_{2}\left(\theta \right)$ on θ∈[0,1] and that $F_{1}^{*}\left(\eta \right)\leq F_{2}^{*}\left(\eta \right)$ on H=[0,2].

We now state a property between dual duo Bregman divergences:

**Property** **2** (Dual duo Fenchel–Young and Bregman divergences)**.** *We have*

(51) $$Y_{F_{1},F_{2}^{*}}\left(\theta :\eta^{\prime }\right)=B_{F_{1},F_{2}}\left(\theta :\theta^{\prime }\right)=B_{F_{2}^{*},F_{1}^{*}}\left(\eta^{\prime }:\eta \right)=Y_{F_{2}^{*},F_{1}}\left(\eta^{\prime }:\theta \right)$$

**Proof.**  From the Fenchel–Young equalities of the inequalities, we have $F_{1}\left(\theta \right)=\theta^{⊤}\eta -F_{1}^{*}\left(\eta \right)$ for $\eta =\nabla F_{1}\left(\theta \right)$ and $F_{2}\left(\theta^{\prime }\right)={\theta^{\prime }}^{⊤}\eta^{\prime }-F_{2}^{*}\left(\eta^{\prime }\right)$ with $\eta^{\prime }=\nabla F_{2}\left(\theta^{\prime }\right)$. Thus we have

(52) $$\begin{array}{ccc} B_{F_{1},F_{2}}\left(\theta :\theta^{\prime }\right) & = & F_{1}\left(\theta \right)-F_{2}\left(\theta^{\prime }\right)-{\left(\theta -\theta^{\prime }\right)}^{⊤}\nabla F_{2}\left(\theta^{\prime }\right), \end{array}$$

(53) $$\begin{array}{ccc} & = & \theta^{⊤}\eta -F_{1}^{*}\left(\eta \right)-{\theta^{\prime }}^{⊤}\eta^{\prime }+F_{2}^{*}\left(\eta^{\prime }\right)-{\left(\theta -\theta^{\prime }\right)}^{⊤}\eta^{\prime }, \end{array}$$

(54) $$\begin{array}{ccc} & = & F_{2}^{*}\left(\eta^{\prime }\right)-F_{1}^{*}\left(\eta \right)-{\left(\eta^{\prime }-\eta \right)}^{⊤}\theta , \end{array}$$

(55) $$\begin{array}{ccc} & = & B_{F_{2}^{*},F_{1}^{*}}\left(\eta^{\prime }:\eta \right). \end{array}$$

Recall that $F_{1}\left(\theta \right)\geq F_{2}\left(\theta \right)$ implies that $F_{1}^{*}\left(\eta \right)\leq F_{2}^{*}\left(\eta \right)$ (Lemma 1), $\theta =\nabla F_{1}^{*}\left(\eta \right)$, and therefore the dual duo Bregman divergence is non-negative:

$$\begin{array}{ccc} B_{F_{2}^{*},F_{1}^{*}}\left(\eta^{\prime }:\eta \right) & = & F_{2}^{*}\left(\eta^{\prime }\right)-F_{1}^{*}\left(\eta \right)-{\left(\eta^{\prime }-\eta \right)}^{⊤}\theta ,\\ & \geq & \underset{B_{F_{1}^{*}}\left(\eta^{\prime }:\eta \right)\geq 0}{\underset{︸}{F_{1}^{*}\left(\eta^{\prime }\right)-F_{1}^{*}\left(\eta \right)-{\left(\eta^{\prime }-\eta \right)}^{⊤}\nabla F_{1}^{*}\left(\eta \right)}}. \end{array}$$

 □

## 4. Kullback–Leibler Divergence between Distributions of Truncated Exponential Families

Let $E_{1}=\left\{P_{\theta }\;:\;\theta \in \Theta_{1}\right\}$ be an exponential family of distributions all dominated by μ with Radon–Nikodym density $p_{\theta }\left(x\right)=\exp\left(\theta^{⊤}t\left(x\right)-F_{1}\left(\theta \right)+k\left(x\right)\right)\;d\mu \left(x\right)$ defined on the support $X_{1}$. Let $E_{2}=\left\{Q_{\theta }\;:\;\theta \in \Theta_{2}\right\}$ be another exponential family of distributions all dominated by μ with Radon–Nikodym density $q_{\theta }\left(x\right)=\exp\left(\theta^{⊤}t\left(x\right)-F_{2}\left(\theta \right)+k\left(x\right)\right)\;d\mu \left(x\right)$ defined on the support $X_{2}$ such that $X_{1}\subseteq X_{2}$. Let ${\tilde{p}}_{\theta }\left(x\right)=\exp\left(\theta^{⊤}t\left(x\right)+k\left(x\right)\right)\;d\mu \left(x\right)$ be the common unnormalized density so that

(56) $$p_{\theta }\left(x\right)=\frac{{\tilde{p}}_{\theta }\left(x\right)}{Z_{1}\left(\theta \right)}$$

and

(57) $$q_{\theta }\left(x\right)=\frac{{\tilde{p}}_{\theta }\left(x\right)}{Z_{2}\left(\theta \right)}=\frac{Z_{1}\left(\theta \right)}{Z_{2}\left(\theta \right)}\;p_{\theta }\left(x\right),$$

with $Z_{1}\left(\theta \right)=\exp\left(F_{1}\left(\theta \right)\right)$ and $Z_{2}\left(\theta \right)=\exp\left(F_{2}\left(\theta \right)\right)$ being the log-normalizer functions of $E_{1}$ and $E_{2}$, respectively.

We have

(58) $$\begin{array}{ccc} D_{\mathrm{KL}}\left[p_{\theta_{1}}:q_{\theta_{2}}\right] & = & \int_{X_{1}}p_{\theta_{1}}\left(x\right)\log\frac{p_{\theta_{1}}\left(x\right)}{q_{\theta_{2}}\left(x\right)}d\mu \left(x\right), \end{array}$$

(59) $$\begin{array}{ccc} & = & \int_{X_{1}}p_{\theta_{1}}\left(x\right)\log\frac{p_{\theta_{1}}\left(x\right)}{p_{\theta_{2}}\left(x\right)}d\mu \left(x\right)+\int_{X_{1}}p_{\theta_{1}}\left(x\right)\log\left(\frac{Z_{2}\left(\theta_{2}\right)}{Z_{1}\left(\theta_{2}\right)}\right)d\mu \left(x\right), \end{array}$$

(60) $$\begin{array}{ccc} & = & D_{\mathrm{KL}}\left[p_{\theta_{1}}:p_{\theta_{2}}\right]+\log Z_{2}\left(\theta_{2}\right)-\log Z_{1}\left(\theta_{2}\right). \end{array}$$

Since $D_{\mathrm{KL}}\left[p_{\theta_{1}}:p_{\theta_{2}}\right]=B_{F_{1}}\left(\theta_{2}:\theta_{1}\right)$ and $\log Z_{i}\left(\theta \right)=F_{i}\left(\theta \right)$, we obtain

(61) $$\begin{array}{ccc} D_{\mathrm{KL}}\left[p_{\theta_{1}}:q_{\theta_{2}}\right] & = & B_{F_{1}}\left(\theta_{2}:\theta_{1}\right)+F_{2}\left(\theta_{2}\right)-F_{1}\left(\theta_{2}\right), \end{array}$$

(62) $$\begin{array}{ccc} & = & F_{1}\left(\theta_{2}\right)-F_{1}\left(\theta_{1}\right)-{\left(\theta_{2}-\theta_{1}\right)}^{⊤}\nabla F_{1}\left(\theta_{1}\right)+F_{2}\left(\theta_{2}\right)-F_{1}\left(\theta_{2}\right), \end{array}$$

(63) $$\begin{array}{ccc} & = & F_{2}\left(\theta_{2}\right)-F_{1}\left(\theta_{1}\right)-{\left(\theta_{2}-\theta_{1}\right)}^{⊤}\nabla F_{1}\left(\theta_{1}\right)=:B_{F_{2},F_{1}}\left(\theta_{2}:\theta_{1}\right). \end{array}$$

Observe that since $X_{1}\subseteq X2$, we have:

(64) $$F_{2}\left(\theta \right)=\log\int_{X_{2}}{\tilde{p}}_{\theta }\left(x\right)\;d\mu \left(x\right)\geq \log\int_{X_{1}}{\tilde{p}}_{\theta }\left(x\right)\;d\mu \left(x\right):=F_{1}\left(\theta \right).$$

Therefore $\Theta_{2}\subseteq \Theta_{1}$, and the common natural parameter space is $\Theta_{12}=\Theta_{1}\cap \Theta_{2}=\Theta_{2}$.

Notice that the reverse Kullback–Leibler divergence $D_{\mathrm{KL}}^{*}\left[p_{\theta_{1}}:q_{\theta_{2}}\right]=D_{\mathrm{KL}}\left[q_{\theta_{2}}:p_{\theta_{1}}\right]=+\infty$ since Qθ2≪Pθ1.

**Theorem** **1** (Kullback–Leibler divergence between truncated exponential family densities)**.** *Let $E_{2}=\left\{q_{\theta_{2}}\right\}$ be an exponential family with support $X_{2}$, and $E_{1}=\left\{p_{\theta_{1}}\right\}$ a truncated exponential family of $E_{2}$ with support $X_{1}\subset X_{2}$. Let $F_{1}$ and $F_{2}$ denote the log-normalizers of $E_{1}$ and $E_{2}$ and $\eta_{1}$ and $\eta_{2}$ the moment parameters corresponding to the natural parameters $\theta_{1}$ and $\theta_{2}$. Then the Kullback–Leibler divergence between a truncated density of $E_{1}$ and a density of $E_{2}$ is*

(65) $$D_{\mathrm{KL}}\left[p_{\theta_{1}}:q_{\theta_{2}}\right]=Y_{F_{2},F_{1}^{*}}\left(\theta_{2}:\eta_{1}\right)=B_{F_{2},F_{1}}\left(\theta_{2}:\theta_{1}\right)=B_{F_{1}^{*},F_{2}^{*}}\left(\eta_{1}:\eta_{2}\right)=Y_{F_{1}^{*},F_{2}}\left(\eta_{1}:\theta_{2}\right).$$

For example, consider the calculation of the KLD between an exponential distribution (view as half a Laplacian distribution, i.e., a truncated Laplacian distribution on the positive real support) and a Laplacian distribution defined on the real line support.

**Example** **4.** *Let $R_{++}=\left\{x\in R\;:\;x>0\right\}$ denote the set of positive reals. Let $E_{1}=\left\{p_{\lambda }\left(x\right)=\lambda \exp\left(-\lambda x\right),\lambda \in R_{++},x>0\right\}$ and $E_{2}=\{q_{\lambda }\left(x\right)=\lambda \exp\left(-\lambda |x|\right),\lambda \in R_{++},x\in R\}$ denote the exponential families of exponential distributions and Laplacian distributions, respectively. We have the sufficient statistic t(x)=−|x| and natural parameter θ=λ so that ${\tilde{p}}_{\theta }\left(x\right)=\exp\left(-|x|\theta \right)$. The log-normalizers are $F_{1}\left(\theta \right)=-\log\theta$ and $F_{2}\left(\theta \right)=-\log\theta +\log2$ (hence $F_{2}\left(\theta \right)\geq F_{1}\left(\theta \right)$). The moment parameter $\eta =\nabla F_{1}\left(\theta \right)=\nabla F_{2}\left(\theta \right)=-\frac{1}{\theta }=-\frac{1}{\lambda }$. Thus using the duo Bregman divergence, we have:*

(66) $$\begin{array}{ccc} D_{\mathrm{KL}}\left[p_{\theta_{1}}:q_{\theta_{2}}\right] & = & B_{F_{2},F_{1}}\left(\theta_{2}:\theta_{1}\right), \end{array}$$

(67) $$\begin{array}{ccc} & = & F_{2}\left(\theta_{2}\right)-F_{1}\left(\theta_{1}\right)-{\left(\theta_{2}-\theta_{1}\right)}^{⊤}\nabla F_{1}\left(\theta_{1}\right), \end{array}$$

(68) $$\begin{array}{ccc} & = & \log2+\log\frac{\lambda_{1}}{\lambda_{2}}+\frac{\lambda_{2}}{\lambda_{1}}-1. \end{array}$$

*Moreover, we can interpret that divergence using the Itakura–Saito divergence [24]:*

(69) $$D_{\mathrm{IS}}\left[\lambda_{1}:\lambda_{2}\right]:=\frac{\lambda_{1}}{\lambda_{2}}-\log\frac{\lambda_{1}}{\lambda_{2}}-1\geq 0.$$

*we have*

(70) $$D_{\mathrm{KL}}\left[p_{\theta_{1}}:q_{\theta_{2}}\right]=D_{\mathrm{IS}}\left[\lambda_{2}:\lambda_{1}\right]+\log2\geq 0.$$

*We check the result using the duo Fenchel–Young divergence:*

(71) $$D_{\mathrm{KL}}\left[p_{\theta_{1}}:q_{\theta_{2}}\right]=Y_{F_{2},F_{1}^{*}}\left(\theta_{2}:\eta_{1}\right),$$

with $F_{1}^{*}\left(\eta \right)=-1+\log\left(-\frac{1}{\eta }\right)$:

(72) $$\begin{array}{ccc} D_{\mathrm{KL}}\left[p_{\theta_{1}}:q_{\theta_{2}}\right] & = & Y_{F_{2},F_{1}^{*}}\left(\theta_{2}:\eta_{1}\right), \end{array}$$

(73) $$\begin{array}{ccc} & = & -\log\lambda_{2}+\log2-1+\log\lambda_{1}+\frac{\lambda_{2}}{\lambda_{1}}, \end{array}$$

(74) $$\begin{array}{ccc} & = & \log\frac{\lambda_{1}}{\lambda_{2}}+\frac{\lambda_{2}}{\lambda_{1}}+\log_{2}-1. \end{array}$$

Next, consider the calculation of the KLD between a half-normal distribution and a (full) normal distribution:

**Example** **5.** *Consider $E_{1}$ and $E_{2}$ to be the scale family of the half standard normal distributions and the scale family of the standard normal distribution, respectively. We have ${\tilde{p}}_{\theta }\left(x\right)=\exp\left(-\frac{x^{2}}{2\sigma^{2}}\right)$ with $Z_{1}\left(\theta \right)=\sigma \sqrt{\frac{\pi }{2}}$ and $Z_{2}\left(\theta \right)=\sigma \sqrt{2\pi }$. Let the sufficient statistic be $t\left(x\right)=-\frac{x^{2}}{2}$ so that the natural parameter is $\theta =\frac{1}{\sigma^{2}}\in R_{++}$. Here, we have both $\Theta_{1}=\Theta_{2}=R_{++}$. For this example, we check that $Z_{1}\left(\theta \right)=\frac{1}{2}\;Z_{2}\left(\theta \right)$. We have $F_{1}\left(\theta \right)=-\frac{1}{2}\log\theta +\frac{1}{2}\log\frac{\pi }{2}$ and $F_{2}\left(\theta \right)=-\frac{1}{2}\log\theta +\frac{1}{2}\log\left(2\pi \right)$ (with $F_{2}\left(\theta \right)\geq F_{1}\left(\theta \right)$). We have $\eta =-\frac{1}{2\theta }=-\frac{1}{2}\sigma^{2}$. The KLD between two half scale normal distributions is*

(75) $$\begin{array}{ccc} D_{\mathrm{KL}}\left[p_{\theta_{1}}:p_{\theta_{2}}\right] & = & B_{F_{1}}\left(\theta_{2}:\theta_{1}\right), \end{array}$$

(76) $$\begin{array}{ccc} & = & \frac{1}{2}\left(\log\frac{\sigma_{2}^{2}}{\sigma_{1}^{2}}+\frac{\sigma_{1}^{2}}{\sigma_{2}^{2}}-1\right). \end{array}$$

*Since $F_{1}\left(\theta \right)$ and $F_{2}\left(\theta \right)$ differ only by a constant and the Bregman divergence is invariant under an affine term of its generator, we have*

(77) $$\begin{array}{ccc} D_{\mathrm{KL}}\left[q_{\theta_{1}}:q_{\theta_{2}}\right] & = & B_{F_{2}}\left(\theta_{2}:\theta_{1}\right), \end{array}$$

(78) $$\begin{array}{ccc} & = & B_{F_{1}}\left(\theta_{2}:\theta_{1}\right)=\frac{1}{2}\left(\log\frac{\sigma_{2}^{2}}{\sigma_{1}^{2}}+\frac{\sigma_{1}^{2}}{\sigma_{2}^{2}}-1\right). \end{array}$$

*Moreover, we can interpret those Bregman divergences as half of the Itakura–Saito divergence:*

(79) $$D_{\mathrm{KL}}\left[p_{\theta_{1}}:p_{\theta_{2}}\right]=D_{\mathrm{KL}}\left[q_{\theta_{1}}:q_{\theta_{2}}\right]=B_{F_{2}}\left(\theta_{2}:\theta_{1}\right)=\frac{1}{2}D_{\mathrm{IS}}\left[\sigma_{1}^{2}:\sigma_{2}^{2}\right].$$

*It follows that*

(80) $$\begin{array}{ccc} D_{\mathrm{KL}}\left[p_{\theta_{1}}:q_{\theta_{2}}\right] & = & B_{F_{2},F_{1}}\left(\theta_{2}:\theta_{1}\right)=F_{2}\left(\theta_{2}\right)-F_{1}\left(\theta_{1}\right)-{\left(\theta_{2}-\theta_{1}\right)}^{⊤}\nabla F_{1}\left(\theta_{1}\right), \end{array}$$

(81) $$\begin{array}{ccc} & = & \frac{1}{2}\left(\log\frac{\sigma_{2}^{2}}{\sigma_{1}^{2}}+\frac{\sigma_{1}^{2}}{\sigma_{2}^{2}}+\log4-1\right), \end{array}$$

(82) $$\begin{array}{ccc} & = & D_{\mathrm{KL}}\left[q_{\theta_{1}}:q_{\theta_{2}}\right]+\log2. \end{array}$$

*Since log2>0, we have $D_{\mathrm{KL}}\left[p_{\theta_{1}}:q_{\theta_{2}}\right]\geq D_{\mathrm{KL}}\left[q_{\theta_{1}}:q_{\theta_{2}}\right]$.*

Thus the Kullback–Leibler divergence between a truncated density and another density of the same exponential family amounts to calculate a duo Bregman divergence on the reverse parameter order: $D_{\mathrm{KL}}\left[p_{\theta_{1}}:q_{\theta_{2}}\right]=B_{F_{2},F_{1}}\left(\theta_{2}:\theta_{1}\right)$. Let $D_{\mathrm{KL}}^{*}\left[p:q\right]:=D_{\mathrm{KL}}\left[q:p\right]$ be the reverse Kullback–Leibler divergence. Then $D_{\mathrm{KL}}^{*}\left[q_{\theta_{2}}:p_{\theta_{1}}\right]=B_{F_{2},F_{1}}\left(\theta_{2}:\theta_{1}\right)$.

Notice that truncated exponential families are also exponential families but those exponential families may be non-steep [25].

Let $E_{1}=\left\{p_{\theta }^{a_{1},b_{1}}\right\}$ and $E_{2}=\left\{p_{\theta }^{a_{2},b_{2}}\right\}$ be two truncated exponential families of the exponential family $E=\{p_{\theta }=\frac{dP_{\theta }}{d\mu }\}$ with log-normalizer F(θ) such that

(83) $$p_{\theta }^{a_{i},b_{i}}\left(x\right)=\frac{p_{\theta }\left(x\right)}{Z_{a_{i},b_{i}}\left(\theta \right)},$$

with $Z_{a_{i},b_{i}}\left(\theta \right)=\Phi_{\theta }\left(b_{i}\right)-\Phi_{\theta }\left(a_{i}\right)$, where $\Phi_{\theta }\left(x\right)$ denotes the CDF of $p_{\theta }\left(x\right)$. Then the log-normalizer of $E_{i}$ is $F_{i}\left(\theta \right)=F\left(\theta \right)+\log\left(\Phi_{\theta }\left(b_{i}\right)-\Phi_{\theta }\left(a_{i}\right)\right)$ for i∈{1,2}.

**Corollary** **1** (Kullback–Leibler divergence between densities of truncated exponential families)**.** *Let $E_{i}=\left\{p_{\theta }^{a_{i},b_{i}}\right\}$ be truncated exponential families of the exponential family $E=\{p_{\theta }\}$ with support $X_{i}=\left[a_{i},b_{i}\right]\subset X$ (where X denotes the support of E) for i∈{1,2}. Then the Kullback–Leibler divergence between $p_{\theta_{1}}^{a_{1},b_{1}}$ and $p_{\theta_{2}}^{a_{2},b_{2}}$ is infinite if [a1,b1]⊂[a2,b2] and has the following formula when $\left[a_{1},b_{1}\right]\subset \left[a_{2},b_{2}\right]$:*

(84) $$D_{\mathrm{KL}}\left[p_{\theta_{1}}^{a_{1},b_{1}}:p_{\theta_{2}}^{a_{2},b_{2}}\right]=D_{\mathrm{KL}}\left[p_{\theta_{1}}^{a_{1},b_{1}}:p_{\theta_{2}}^{a_{1},b_{1}}\right]+\log\frac{Z_{a_{2},b_{2}}\left(\theta_{2}\right)}{Z_{a_{1},b_{1}}\left(\theta_{2}\right)}.$$

**Proof.**  We have $p_{\theta }^{a_{1},b_{1}}=\frac{p_{\theta }}{Z_{a_{1},b_{1}}\left(\theta \right)}$ and $p_{\theta }^{a_{2},b_{2}}=\frac{p_{\theta }}{Z_{a_{2},b_{2}}\left(\theta \right)}$. Therefore $p_{\theta }^{a_{2},b_{2}}=p_{\theta }^{a_{1},b_{1}}\frac{Z_{a_{1},b_{1}}\left(\theta \right)}{Z_{a_{2},b_{2}}\left(\theta \right)}$. Thus we have

(85) $$\begin{array}{ccc} D_{\mathrm{KL}}\left[p_{\theta_{1}}^{a_{1},b_{1}}:p_{\theta_{2}}^{a_{2},b_{2}}\right] & = & \int_{X_{1}}p_{\theta_{1}}^{a_{1},b_{1}}\left(x\right)\log\frac{p_{\theta_{1}}^{a_{1},b_{1}}\left(x\right)}{p_{\theta_{2}}^{a_{2},b_{2}}}d\mu \left(x\right), \end{array}$$

(86) $$\begin{array}{ccc} & = & \int_{X_{1}}p_{\theta_{1}}^{a_{1},b_{1}}\left(x\right)\log\frac{p_{\theta_{1}}^{a_{1},b_{1}}\left(x\right)}{p_{\theta_{2}}^{a_{1},b_{1}}}d\mu \left(x\right)+\log\frac{Z_{a_{2},b_{2}}\left(\theta_{2}\right)}{Z_{a_{1},b_{1}}\left(\theta_{2}\right)}, \end{array}$$

(87) $$\begin{array}{ccc} & = & D_{\mathrm{KL}}\left[p_{\theta_{1}}^{a_{1},b_{1}}:p_{\theta_{2}}^{a_{1},b_{1}}\right]+\log\frac{Z_{a_{2},b_{2}}\left(\theta_{2}\right)}{Z_{a_{1},b_{1}}\left(\theta_{2}\right)}. \end{array}$$

 □

Thus the KLD between truncated exponential family densities $p_{\theta_{1}}^{a_{1},b_{1}}$ and $p_{\theta_{2}}^{a_{2},b_{2}}$ amounts to the KLD between the densities with the same truncation parameter with an additive term depending on the log ratio of the mass with respect to the truncated supports evaluated at $\theta_{2}$. We shall illustrate with two examples the calculation of the KLD between truncated exponential families.

**Example** **6.** *Consider the calculation of the KLD between a truncated exponential distribution $p_{\lambda_{1}}^{a_{1},b_{1}}$ with support $X_{1}=\left[a_{1},b_{1}\right]$ ($b_{1}>a_{1}\geq 0$) and another truncated exponential distribution $p_{\lambda_{2}}^{a_{2},b_{2}}$ with support $X_{2}=\left[a_{2},b_{2}\right]$ ($b_{2}>a_{2}\geq 0$). We have $p_{\lambda }\left(x\right)=\lambda \exp\left(-\lambda x\right)$ (density of the untruncated exponential family with natural parameter θ=λ, sufficient statistic t(x)=−x and log-normalizer F(θ)=−logθ), $p_{\lambda_{1}}^{a_{1},b_{1}}=\frac{1}{Z_{a_{1},b_{1}}\left(\lambda \right)}p_{\lambda_{1}}\left(x\right)$, and $p_{\lambda_{2}}^{a_{2},b_{2}}=\frac{1}{Z_{a_{2},b_{2}}\left(\lambda \right)}p_{\lambda_{2}}\left(x\right)$. Let $\Phi_{\lambda }\left(x\right)=1-\exp\left(-\lambda x\right)$ denote the cumulative distribution function of the exponential distribution. We have $Z_{a,b}\left(\lambda \right)=\Phi_{b}\left(\lambda \right)-\Phi_{a}\left(\lambda \right)$ and*

(88) $$F_{a,b}\left(\lambda \right)=F\left(\lambda \right)+\log\left(\Phi_{b}\left(\lambda \right)-\Phi_{a}\left(\lambda \right)\right)=-\log\lambda +\log\left(e^{-\lambda a}-e^{-\lambda b}\right).$$

*If $\left[a_{1},b_{1}\right]\notin \left[a_{2},b_{2}\right]$ then $D_{\mathrm{KL}}\left[p_{\lambda_{1}}:q_{\lambda_{2}}\right]=+\infty$. Otherwise, $\left[a_{1},b_{1}\right]\in \left[a_{2},b_{2}\right]$, and the exponential family $\left\{p_{\lambda }\right\}$ is the truncated exponential family $\left\{q_{\lambda }\right\}$. Using the computer algebra system Maxima (https://maxima.sourceforge.io/ accessed on 15 March 2022), we find that*

(89) $$-E_{p_{\lambda }}\left[x\right]=\frac{\left(1+\lambda b\right)e^{\lambda a}-\left(1+\lambda a\right)e^{\lambda b}}{\lambda (e^{\lambda b}-e^{\lambda a})}=F_{a,b}^{\prime }\left(\lambda \right).$$

*Thus we have:*

(90) $$\begin{array}{ccc} D_{\mathrm{KL}}\left[p_{\lambda_{1}}^{a_{1},b_{1}}:q_{\lambda_{2}}^{a_{2},b_{2}}\right] & = & B_{F_{2},F_{1}}\left(\theta_{2}:\theta_{1}\right),\\ & = & F_{a_{2},b_{2}}\left(\lambda_{2}\right)-F_{a_{1},b_{1}}\left(\lambda_{1}\right)-\left(\lambda_{2}-\lambda_{1}\right)F_{a_{1},b_{1}}^{\prime }\left(\lambda_{1}\right), \end{array}$$

(91) $$\begin{array}{ccc} & = & \log\frac{\lambda_{1}}{\lambda_{2}}+\left(\lambda_{2}-\lambda_{1}\right)\;E_{p_{\lambda_{1}}}\left[x\right]+\log\frac{e^{-\lambda_{2}a_{2}}-e^{-\lambda_{2}b_{2}}}{e^{-\lambda_{1}a_{1}}-e^{-\lambda_{1}b_{1}}}. \end{array}$$

*When $a_{1}=a_{2}=0$ and $b_{1}=b_{2}=+\infty$, we recover the KLD between two exponential distributions $p_{\lambda_{1}}$ and $p_{\lambda_{2}}$:*

(92) $$\begin{array}{ccc} D_{\mathrm{KL}}\left[p_{\lambda_{1}}:p_{\lambda_{2}}\right] & = & B_{F}\left(\lambda_{2}:\lambda_{1}\right), \end{array}$$

(93) $$\begin{array}{ccc} & = & F\left(\theta_{2}\right)-F\left(\theta_{1}\right)-\left(\theta_{2}-\theta_{1}\right)F^{\prime }\left(\theta_{1}\right), \end{array}$$

(94) $$\begin{array}{ccc} & = & \frac{\lambda_{2}}{\lambda_{1}}-\log\frac{\lambda_{2}}{\lambda_{1}}-1=D_{\mathrm{IS}}\left[\lambda_{2}:\lambda_{1}\right]. \end{array}$$

*Note that the KLD between two truncated exponential distributions with the same truncation support X=[a,b] is*

(95) $$D_{\mathrm{KL}}\left[p_{\lambda_{1}}^{a,b}:p_{\lambda_{2}}^{a,b}\right]=\log\frac{\lambda_{2}}{\lambda_{1}}+\log\frac{\Phi_{\lambda_{2}}\left(b\right)-\Phi_{\lambda_{2}}\left(a\right)}{\Phi_{\lambda_{1}}\left(b\right)-\Phi_{\lambda_{1}}\left(a\right)}+\left(\lambda_{2}-\lambda_{1}\right)E_{p_{1}^{a,b}}\left[x\right].$$

*We also check Corollary 1:*

(96) $$D_{\mathrm{KL}}\left[p_{\lambda_{1}}^{a_{1},b_{1}}:p_{\lambda_{2}}^{a_{2},b_{2}}\right]=D_{\mathrm{KL}}\left[p_{\lambda_{1}}^{a_{1},b_{1}}:p_{\lambda_{2}}^{a_{1},b_{1}}\right]+\log\frac{Z_{a_{2},b_{2}}\left(\lambda_{2}\right)}{Z_{a_{1},b_{1}}\left(\lambda_{2}\right)},$$

The next example shows how to compute the Kullback–Leibler divergence between two truncated normal distributions:

**Example** **7.** *Let $N_{a,b}\left(m,s\right)$ denote a truncated normal distribution with support the open interval (a,b) (a<b) and probability density function defined by:*

(97) $$p_{m,s}^{a,b}\left(x\right)=\frac{1}{Z_{a,b}\left(m,s\right)}\;\exp\left(-\frac{{\left(x-m\right)}^{2}}{2s^{2}}\right),$$

*where $Z_{a,b}\left(m,s\right)$ is related to the partition function [26] expressed using the cumulative distribution function (CDF) $\Phi_{m,s}\left(x\right)$:*

(98) $$Z_{a,b}\left(m,s\right)=\sqrt{2\pi }s\;\left(\Phi_{m,s}\left(b\right)-\Phi_{m,s}\left(a\right)\right),$$

*with*

(99) $$\Phi_{m,s}\left(x\right)=\frac{1}{2}\left(1+\mathrm{erf}\left(\frac{x-m}{\sqrt{2}s}\right)\right),$$

*where erf(x) is the error function:*

(100) $$\mathrm{erf}\left(x\right):=\frac{2}{\sqrt{\pi }}\;\int_{0}^{x}e^{-t^{2}}dt.$$

*Thus we have $\mathrm{erf}\left(x\right)=2\;\Phi \left(\sqrt{2}x\right)-1$ where $\Phi \left(x\right)=\Phi_{0,1}\left(x\right)$.*

*The pdf can also be written as*

(101) $$p_{m,s}^{a,b}\left(x\right)=\frac{1}{s}\frac{\phi (\frac{x-m}{s})}{\Phi \left(\frac{b-m}{s}\right)-\Phi \left(\frac{a-m}{s}\right)},$$

*where ϕ(x) denotes the standard normal pdf ($\phi \left(x\right)=p_{0,1}^{-\infty ,+\infty }\left(x\right)$):*

(102) $$\phi \left(x\right):=\frac{1}{\sqrt{2\pi }}\;\exp\left(-\frac{x^{2}}{2}\right),$$

*and $\Phi \left(x\right)=\Phi_{0,1}\left(x\right)=\int_{-\infty }^{x}\phi \left(t\right)\;dt$ is the standard normal CDF. When a=−∞ and b=+∞, we have $Z_{-\infty ,\infty }\left(m,s\right)=\sqrt{2\pi }\;s$ since Φ(−∞)=0 and Φ(+∞)=1.*

*The density $p_{m,s}^{a,b}\left(x\right)$ belongs to an exponential family $E_{a,b}$ with natural parameter $\theta =\left(\frac{m}{s^{2}},-\frac{1}{2s^{2}}\right)$, sufficient statistics $t\left(x\right)=\left(x,x^{2}\right)$, and log-normalizer:*

(103) $$F_{a,b}\left(\theta \right)=-\frac{\theta_{1}^{2}}{4\theta_{2}}+\log Z_{a,b}\left(\theta \right)$$

*The natural parameter space is $\Theta =R\times R_{--}$ where $R_{--}=\left\{x\in R\;:\;x<0\right\}$ denotes the set of negative real numbers.*

*The log-normalizer can be expressed using the source parameters (m,s) (which are not the mean and standard deviation when the support is truncated, hence the notation m and s):*

(104) $$\begin{array}{ccc} F_{a,b}\left(m,s\right) & = & \frac{m^{2}}{2s^{2}}+\log Z_{a,b}\left(m,s\right), \end{array}$$

(105) $$\begin{array}{ccc} & = & \frac{m^{2}}{2s^{2}}+\frac{1}{2}\log2\pi s^{2}+\log\left(\Phi_{m,s}\left(b\right)-\Phi_{m,s}\left(a\right)\right). \end{array}$$

*We shall use the fact that the gradient of the log-normalizer of any exponential family distribution amounts to the expectation of the sufficient statistics [1]:*

(106) $$\nabla F_{a,b}\left(\theta \right)=E_{p_{m,s}^{a,b}}\left[t\left(x\right)\right]=\eta .$$

*Parameter η is called the moment or expectation parameter [1].*

*The mean $\mu \left(m,s;a,b\right)=E_{p_{m,s}^{a,b}}\left[x\right]=\frac{\partial }{\partial \theta_{1}}F_{a,b}\left(\theta \right)$ and the variance $\sigma^{2}\left(m,s;a,b\right)=E_{p_{m,s}^{a,b}}\left[x^{2}\right]-\mu^{2}$ (with $E_{p_{m,s}^{a,b}}\left[x^{2}\right]=\frac{\partial }{\partial \theta_{2}}F_{a,b}\left(\theta \right)$) of the truncated normal $p_{m,s}^{a,b}$ can be expressed using the following formula [26,27] (page 25):*

(107) $$\begin{array}{ccc} \mu (m,s;a,b) & = & m-s\;\frac{\phi (\beta )-\phi (\alpha )}{\Phi (\beta )-\Phi (\alpha )}, \end{array}$$

(108) $$\begin{array}{ccc} \sigma^{2}\left(m,s;a,b\right) & = & s^{2}\;\left(1-\frac{\beta \phi (\beta )-\alpha \phi (\alpha )}{\Phi (\beta )-\Phi (\alpha )}-{\left(\frac{\phi (\beta )-\phi (\alpha )}{\Phi (\beta )-\Phi (\alpha )}\right)}^{2}\right), \end{array}$$

*where $\alpha :=\frac{a-m}{s}$ and $\beta :=\frac{b-m}{s}$. Thus we have the following moment parameter $\eta =(\eta_{1},\eta_{2})$ with*

(109) $$\begin{array}{ccc} \eta_{1}\left(m,s;a,b\right) & = & E_{p_{m,s}^{a,b}}\left[x\right]=\mu \left(m,s;a,b\right), \end{array}$$

(110) $$\begin{array}{ccc} \eta_{2}\left(m,s;a,b\right) & = & E_{p_{m,s}^{a,b}}\left[x^{2}\right]=\sigma^{2}\left(m,s;a,b\right)+\mu^{2}\left(m,s;a,b\right). \end{array}$$

*Now consider two truncated normal distributions $p_{m_{1},s_{1}}^{a_{1},b_{1}}$ and $p_{m_{2},s_{2}}^{a_{2},b_{2}}$ with $\left[a_{1},b_{1}\right]\subseteq \left[a_{2},b_{2}\right]$ (otherwise, we have $D_{\mathrm{KL}}\left[p_{m_{1},s_{1}}^{a_{1},b_{1}}:p_{m_{2},s_{2}}^{a_{2},b_{2}}\right]=+\infty$). Then the KLD between $p_{m_{1},s_{1}}^{a_{1},b_{1}}$ and $p_{m_{2},s_{2}}^{a_{2},b_{2}}$ is equivalent to a duo Bregman divergence:*

(111) $$\begin{array}{ccc} D_{\mathrm{KL}}\left[p_{m_{1},s_{1}}^{a_{1},b_{1}}:p_{m_{2},s_{2}}^{a_{2},b_{2}}\right] & = & F_{m_{2},s_{2}}\left(\theta_{2}\right)-F_{m_{1},s_{1}}\left(\theta_{1}\right)-{\left(\theta_{2}-\theta_{1}\right)}^{⊤}\nabla F_{m_{1},s_{1}}\left(\theta_{1}\right),\\ & = & \frac{m_{2}}{2s_{2}^{2}}-\frac{m_{1}}{2s_{1}^{2}}+\log\frac{Z_{a_{2},b_{2}}\left(m_{2},s_{2}\right)}{Z_{a_{1},b_{1}}\left(m_{1},s_{1}\right)}-\left(\frac{m_{2}}{s_{2}^{2}}-\frac{m_{1}}{s_{1}^{2}}\right)\eta_{1}\left(m_{1},s_{1};a_{1},b_{1}\right)\\ & & -\left(\frac{1}{2s_{1}^{2}}-\frac{1}{2s_{2}^{2}}\right)\eta_{2}\left(m_{1},s_{1};a_{1},b_{1}\right). \end{array}$$

*Note that $F_{m_{2},s_{2}}\left(\theta \right)\geq F_{m_{1},s_{1}}\left(\theta \right)$.*

*This formula is valid for (1) the KLD between two truncated normal distributions, or for (2) the KLD between a truncated normal distribution and a (full support) normal distribution. Note that the formula depends on the erf function used in function Φ. Furthermore, when $a_{1}=a_{2}=-\infty$ and $b_{1}=b_{2}=+\infty$, we recover (3) the KLD between two univariate normal distributions, since $\log\frac{Z_{a_{2},b_{2}}\left(m_{2},s_{2}\right)}{Z_{a_{1},b_{1}}\left(m_{1},s_{1}\right)}=\log\frac{\sigma_{2}}{\sigma_{1}}=\frac{1}{2}\log\frac{\sigma_{2}^{2}}{\sigma_{1}^{2}}$:*

(112) $$D_{\mathrm{KL}}\left[p_{m_{1},s_{1}}:p_{m_{2},s_{2}}\right]=\frac{1}{2}\;\left(\log\frac{s_{2}^{2}}{s_{1}^{2}}+\frac{\sigma_{1}^{2}}{\sigma_{2}^{2}}+\frac{{\left(m_{2}-m_{1}\right)}^{2}}{s_{2}^{2}}-1.\right).$$

*Note that for full support normal distributions, we have μ(m,s;−∞;+∞)=m and $\sigma^{2}\left(m,s;-\infty ;+\infty \right)=s^{2}$.*

The entropy of a truncated normal distribution (an exponential family [28]) is $h\left[p_{m,s}^{a,b}\right]=-\int_{a}^{b}p_{m,s}^{a,b}\left(x\right)\log p_{m,s}^{a,b}dx=-F^{*}\left(\eta \right)=F\left(\theta \right)-\theta^{⊤}\eta$. We find that

(113) $$h\left[p_{m,s}^{a,b}\right]=\log\left(\sqrt{2\pi e}s\;\left(\Phi (\beta )-\Phi (\alpha )\right)\right)+\frac{\alpha \phi (\alpha )-\beta \phi (\beta )}{2\left(\Phi (\beta )-\Phi (\alpha )\right)}.$$

*When (a,b)=(−∞,∞), we have Φ(β)−Φ(α)=1 and αϕ(α)−βϕ(β)=0 since β=−α, ϕ(−x)=ϕ(x) (an even function), and $\lim_{\beta \to +\infty }\beta \phi \left(\beta \right)=0$. Thus we recover the differential entropy of a normal distribution: $h\left[p_{\mu ,\sigma }\right]=\log\left(\sqrt{2\pi e}\sigma \right)$.*

## 5. Bhattacharyya Skewed Divergence between Truncated Densities of an Exponential Family

The Bhattacharyya α-skewed divergence [29,30] between two densities p(x) and q(x) with respect to μ is defined for a skewing scalar parameter α∈(0,1) as:

(114) $$D_{\mathrm{Bhat},\alpha }\left[p:q\right]:=-\log\int_{X}p{\left(x\right)}^{\alpha }q{\left(x\right)}^{1-\alpha }\;d\mu \left(x\right),$$

where X denotes the support of the distributions. The Bhattacharyya distance is

(115) $$D_{\mathrm{Bhat}}\left[p,q\right]=D_{\mathrm{Bhat},\frac{1}{2}}\left[p:q\right]=-\log\int_{X}\sqrt{p(x)q(x)}\;d\mu \left(x\right).$$

The Bhattacharyya distance is not a metric distance since it does not satisfy the triangle inequality. The Bhattacharyya distance is related to the Hellinger distance [31] as follows:

(116) $$D_{H}\left[p,q\right]=\sqrt{\frac{1}{2}\int_{X}{\left(\sqrt{p(x)}-\sqrt{q(x)}\right)}^{2}\;d\mu \left(x\right)}=\sqrt{1-\exp(-D_{\mathrm{Bhat}}\left[p,q\right])}.$$

The Hellinger distance is a metric distance.

Let $I_{\alpha }\left[p:q\right]:=\int_{X}p{\left(x\right)}^{\alpha }q{\left(x\right)}^{1-\alpha }\;d\mu \left(x\right)$ denote the skewed affinity coefficient so that $D_{\mathrm{Bhat},\alpha }\left[p:q\right]=-\log I_{\alpha }\left[p:q\right]$. Since $I_{\alpha }\left[p:q\right]=I_{1-\alpha }\left[q:p\right]$, we have $D_{\mathrm{Bhat},\alpha }\left[p:q\right]=D_{\mathrm{Bhat},1-\alpha }\left[q:p\right]$.

Consider an exponential family $E=\{p_{\theta }\}$ with log-normalizer F(θ). Then it is well-known that the α-skewed Bhattacharyya divergence between two densities of an exponential family amounts to a skewed Jensen divergence [30] (originally called Jensen difference in [32]):

(117) $$D_{\mathrm{Bhat},\alpha }\left[p_{\theta_{1}}:p_{\theta_{2}}\right]=J_{F,\alpha }\left(\theta_{1}:\theta_{2}\right),$$

where the skewed Jensen divergence is defined by

(118) $$J_{F,\alpha }\left(\theta_{1}:\theta_{2}\right)=\alpha F\left(\theta_{1}\right)+\left(1-\alpha \right)F\left(\theta_{2}\right)-F\left(\alpha \theta_{1}+\left(1-\alpha \right)\theta_{2}\right).$$

The convexity of the log-normalizer F(θ) ensures that $J_{F,\alpha }\left(\theta_{1}:\theta_{2}\right)\geq 0$. The Jensen divergence can be extended to full real α by rescaling it by $\frac{1}{\alpha (1-\alpha )}$, see [33].

**Remark** **1.** *The Bhattacharyya skewed divergence $D_{\mathrm{Bhat},\alpha }\left[p:q\right]$ appears naturally as the negative of the log-normalizer of the exponential family induced by the exponential arc $\left\{r_{\alpha }\left(x\right)\;\alpha \in \left(0,1\right)\right\}$ linking two densities p and q with $r_{\alpha }\left(x\right)\propto p{\left(x\right)}^{\alpha }q{\left(x\right)}^{1-\alpha }$. This arc is an exponential family of order 1:*

(119) $$\begin{array}{ccc} r_{\alpha }\left(x\right) & = & \exp\left(\alpha \log p\left(x\right)+\left(1-\alpha \right)\log q\left(x\right)-\log Z_{\alpha }\left(p:q\right)\right), \end{array}$$

(120) $$\begin{array}{ccc} & = & \exp\left(\alpha \log\frac{p(x)}{q(x)}-F_{pq}\left(\alpha \right)\right)\;q\left(x\right). \end{array}$$

*The sufficient statistic is $t\left(x\right)=\frac{p(x)}{q(x)}$, the natural parameter α∈(0,1), and the log-normalizer $F_{pq}\left(\alpha \right)=\log Z_{\alpha }\left(p:q\right)=\log\int p{\left(x\right)}^{\alpha }q{\left(x\right)}^{1-\alpha }d\mu \left(x\right)=-D_{\mathrm{Bhat},\alpha }\left[p:q\right]$. This shows that $D_{\mathrm{Bhat},\alpha }\left[p:q\right]$ is concave with respect to α since log-normalizers $F_{pq}\left(\alpha \right)$ are always convex. Grünwald called those exponential families the likelihood ratio exponential families [34].*

Now, consider calculating $D_{\mathrm{Bhat},\alpha }\left[p_{\theta_{1}}:q_{\theta_{2}}\right]$ where $p_{\theta_{1}}\in E_{1}$ with $E_{1}$ a truncated exponential family of $E_{2}$ and $q_{\theta_{2}}\in E_{2}=\left\{q_{\theta }\right\}$. We have $q_{\theta }\left(x\right)=\frac{Z_{1}\left(\theta \right)}{Z_{2}\left(\theta \right)}p_{\theta }\left(x\right)$, where $Z_{1}\left(\theta \right)$ and $Z_{2}\left(\theta \right)$ are the partition functions of $E_{1}$ and $E_{2}$, respectively. Thus we have

(121) $$I_{\alpha }\left[p_{\theta_{1}}:q_{\theta_{2}}\right]={\left(\frac{Z_{1}\left(\theta_{2}\right)}{Z_{2}\left(\theta_{2}\right)}\right)}^{1-\alpha }\;I_{\alpha }\left[p_{\theta_{1}}:p_{\theta_{2}}\right],$$

and the α-skewed Bhattacharyya divergence is

(122) $$D_{\mathrm{Bhat},\alpha }\left[p_{\theta_{1}}:q_{\theta_{2}}\right]=D_{\mathrm{Bhat},\alpha }\left[p_{\theta_{1}}:p_{\theta_{2}}\right]-\left(1-\alpha \right)\left(F_{1}\left(\theta_{2}\right)-F_{2}\left(\theta_{2}\right)\right).$$

Therefore we obtain

(123) $$\begin{array}{ccc} D_{\mathrm{Bhat},\alpha }\left[p_{\theta_{1}}:q_{\theta_{2}}\right] & = & J_{F_{1},\alpha }\left(\theta_{1}:\theta_{2}\right)-\left(1-\alpha \right)\left(F_{1}\left(\theta_{2}\right)-F_{2}\left(\theta_{2}\right)\right), \end{array}$$

(124) $$\begin{array}{ccc} & = & \alpha F_{1}\left(\theta_{1}\right)+\left(1-\alpha \right)F_{2}\left(\theta_{2}\right)-F_{1}\left(\alpha \theta_{1}+\left(1-\alpha \right)\theta_{2}\right), \end{array}$$

(125) $$\begin{array}{ccc} & =: & J_{F_{1},F_{2},\alpha }\left(\theta_{1}:\theta_{2}\right). \end{array}$$

We call $J_{F_{1},F_{2},\alpha }\left(\theta_{1}:\theta_{2}\right)$ the duo Jensen divergence. Since $F_{2}\left(\theta \right)\geq F_{1}\left(\theta \right)$, we check that

(126) $$J_{F_{1},F_{2},\alpha }\left(\theta_{1}:\theta_{2}\right)\geq J_{F_{1},\alpha }\left(\theta_{1}:\theta_{2}\right)\geq 0.$$

**Figure 7:**
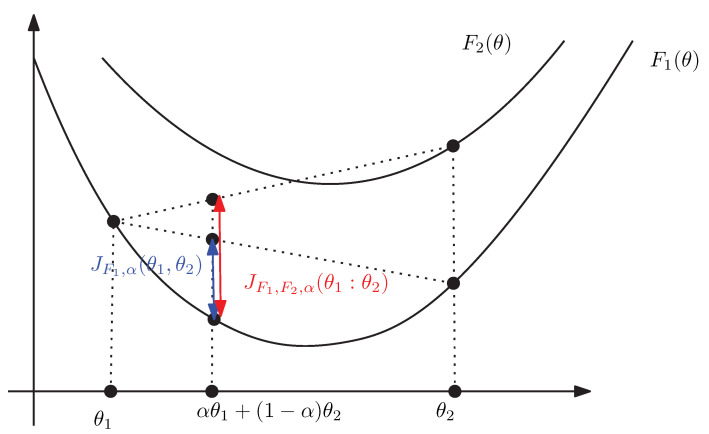
The duo Jensen divergence $J_{F_{1},F_{2},\alpha }\left(\theta_{1}:\theta_{2}\right)$ is greater than the Jensen divergence $J_{F_{1},\alpha }\left(\theta_{1}:\theta_{2}\right)$ for $F_{2}\left(\theta \right)\geq F_{1}\left(\theta \right)$.

Figure 7 illustrates graphically the duo Jensen divergence $J_{F_{1},F_{2},\alpha }\left(\theta_{1}:\theta_{2}\right)$.

**Theorem** **2.** *The α-skewed Bhattacharyya divergence for α∈(0,1) between a truncated density of $E_{1}$ with log-normalizer $F_{1}\left(\theta \right)$ and another density of an exponential family $E_{2}$ with log-normalizer $F_{2}\left(\theta \right)$ amounts to a duo Jensen divergence:*

(127) $$D_{\mathrm{Bhat},\alpha }\left[p_{\theta_{1}}:q_{\theta_{2}}\right]=J_{F_{1},F_{2},\alpha }\left(\theta_{1}:\theta_{2}\right),$$

*where $J_{F_{1},F_{2},\alpha }\left(\theta_{1}:\theta_{2}\right)$ is the duo skewed Jensen divergence induced by two strictly convex functions $F_{1}\left(\theta \right)$ and $F_{2}\left(\theta \right)$ such that $F_{2}\left(\theta \right)\geq F_{1}\left(\theta \right)$:*

(128) $$J_{F_{1},F_{2},\alpha }\left(\theta_{1}:\theta_{2}\right)=\alpha F_{1}\left(\theta_{1}\right)+\left(1-\alpha \right)F_{2}\left(\theta_{2}\right)-F_{1}\left(\alpha \theta_{1}+\left(1-\alpha \right)\theta_{2}\right).$$

In [30], it is reported that

(129) $$\begin{array}{ccc} D_{\mathrm{KL}}\left[p_{\theta_{1}}:p_{\theta_{2}}\right] & = & B_{F}\left(\theta_{2}:\theta_{1}\right), \end{array}$$

(130) $$\begin{array}{ccc} & = & \lim_{\alpha \to 0}\frac{1}{\alpha }J_{F,\alpha }\left(\theta_{2}:\theta_{1}\right)=\lim_{\alpha \to 0}\frac{1}{\alpha }J_{F,1-\alpha }\left(\theta_{1}:\theta_{2}\right), \end{array}$$

(131) $$\begin{array}{ccc} & = & \lim_{\alpha \to 0}\frac{1}{\alpha }D_{\mathrm{Bhat},\alpha }\left[p_{\theta_{2}}:p_{\theta_{1}}\right]=\lim_{\alpha \to 0}\frac{1}{\alpha }D_{\mathrm{Bhat},1-\alpha }\left[p_{\theta_{1}}:p_{\theta_{2}}\right]. \end{array}$$

Indeed, using the first-order Taylor expansion of

(132) $$F\left(\theta_{1}+\alpha \left(\theta_{2}-\theta_{1}\right)\right)\underset{\alpha \to 0}{\approx }F\left(\theta_{1}\right)+\alpha {\left(\theta_{2}-\theta_{1}\right)}^{⊤}\nabla F\left(\theta_{1}\right)$$

when α→0, we check that we have

(133) $$\begin{array}{ccc} \frac{1}{\alpha }J_{F,\alpha }\left(\theta_{2}:\theta_{1}\right) & := & \frac{F\left(\theta_{1}\right)+\alpha \left(F\left(\theta_{2}\right)-F\left(\theta_{1}\right)\right)-F\left(\theta_{1}+\alpha \left(\theta_{2}-\theta_{1}\right)\right)}{\alpha }, \end{array}$$

(134) ≈α→0Equation(132)F(θ1)+α(F(θ2)−F(θ1))−F(θ1)−α(θ2−θ1)⊤∇F(θ1)α,

(135) $$\begin{array}{ccc} & = & F\left(\theta_{2}\right)-F\left(\theta_{1}\right)-{\left(\theta_{2}-\theta_{1}\right)}^{⊤}\nabla F\left(\theta_{1}\right), \end{array}$$

(136) $$\begin{array}{ccc} & =: & B_{F}\left(\theta_{2}:\theta_{1}\right). \end{array}$$

Thus we have $\lim_{\alpha \to 0}\frac{1}{\alpha }J_{F,\alpha }\left(\theta_{2}:\theta_{1}\right)=B_{F}\left(\theta_{2}:\theta_{1}\right)$.

Moreover, we have

(137) $$\lim_{\alpha \to 0}\frac{1}{\alpha }D_{\mathrm{Bhat},1-\alpha }\left[p:q\right]=D_{\mathrm{KL}}\left[p:q\right].$$

Similarly, we can prove that

(138) $$\lim_{\alpha \to 1}\frac{1}{1-\alpha }J_{F_{1},F_{2},\alpha }\left(\theta_{1}:\theta_{2}\right)=B_{F_{2},F_{1}}\left(\theta_{2}:\theta_{1}\right),$$

which can be reinterpreted as

(139) $$\lim_{\alpha \to 1}\frac{1}{1-\alpha }D_{\mathrm{Bhat},\alpha }\left[p_{\theta_{1}}:q_{\theta_{2}}\right]=D_{\mathrm{KL}}\left[p_{\theta_{1}}:q_{\theta_{2}}\right].$$

## 6. Concluding Remarks

We considered the Kullback–Leibler divergence between two parametric densities $p_{\theta }\in E_{1}$ and $q_{\theta^{\prime }}\in E_{2}$ belonging to truncated exponential families [7] $E_{1}$ and $E_{2}$, and we showed that their KLD is equivalent to a duo Bregman divergence on swapped parameter order (Theorem 1). This result generalizes the study of Azoury and Warmuth [13]. The duo Bregman divergence can be rewritten as a duo Fenchel–Young divergence using mixed natural/moment parameterizations of the exponential family densities (Definition 1). This second result generalizes the approach taken in information geometry [15,35]. We showed how to calculate the Kullback–Leibler divergence between two truncated normal distributions as a duo Bregman divergence. More generally, we proved that the skewed Bhattacharyya distance between two parametric densities of truncated exponential families amounts to a duo Jensen divergence (Theorem 2). We showed asymptotically that scaled duo Jensen divergences tend to duo Bregman divergences generalizing a result of [30,33]. This study of duo divergences induced by pair of generators was motivated by the formula obtained for the Kullback–Leibler divergence between two densities of two different exponential families originally reported in [23] (Equation (29)).

It is interesting to find applications of the duo Fenchel–Young, Bregman, and Jensen divergences beyond the scope of calculating statistical distances between truncated exponential family densities. Note that in [36], the authors exhibit a relationship between densities with nested supports and quasi-convex Bregman divergences. However, those considered parametric densities are not exponential families since their supports depend on the parameter. Recently, Khan and Swaroop [37] used this duo Fenchel–Young divergence in machine learning for knowledge-adaptation priors in the so-called change regularizer task.

## Figures and Tables

**Table 1:** Canonical decomposition of the Poisson and the geometric discrete exponential families.

Quantity	Poisson Family P	Geometric Family Q
support	N∪{0}	N∪{0}
base measure	counting measure	counting measure
ordinary parameter	rate λ>0	success probability p∈(0,1)
pmf	$\frac{\lambda^{x}}{x!}\exp\left(-\lambda \right)$	${\left(1-p\right)}^{x}p$
sufficient statistic	$t_{P}\left(x\right)=x$	$t_{Q}\left(x\right)=x$
natural parameter	θ(λ)=logλ	θ(p)=log(1−p)
cumulant function	$F_{P}\left(\theta \right)=\exp\left(\theta \right)$	$F_{Q}\left(\theta \right)=-\log\left(1-\exp\left(\theta \right)\right)$
	$F_{P}\left(\lambda \right)=\lambda$	$F_{Q}\left(p\right)=-\log\left(p\right)$
auxiliary term	$k_{P}\left(x\right)=-\log x!$	$k_{Q}\left(x\right)=0$
moment η=E[t(x)]	η=λ	$\eta =\frac{e^{\theta }}{1-e^{\theta }}=\frac{1}{p}-1$
negentropy	$F_{P}^{*}\left(\eta \left(\lambda \right)\right)=\lambda \log\lambda -\lambda$	$F_{Q}^{*}\left(\eta \left(p\right)\right)=\left(1-\frac{1}{p}\right)\log\left(1-p\right)+\log p$
($F^{*}\left(\eta \right)=\theta \cdot \eta -F\left(\theta \right)$)		

## Data Availability

Not applicable.
